# Placental alkaline phosphatase promotes lipid accumulation and fibrosis in ligamentum flavum hypertrophy and represents a potential target for statin repurposing

**DOI:** 10.3389/fphar.2026.1910904

**Published:** 2026-09-29

**Authors:** Junjie Cheng, Konghe Hu, Jianjun Li, Huanbin Lin, Luqi Zhu, Runfan Chen, Shaoxiong Min, Yang Duan

**Affiliations:** 1 Department of Spine Surgery, Zhujiang Hospital, Southern Medical University, Guangzhou, Guangdong, China; 2 Department of Spine Surgery, The Affiliated Yuebei People’s Hospital of Shantou University Medical College, Shaoguan, Guangdong, China; 3 The Second School of Clinical Medicine, Zhujiang Hospital, Southern Medical University, Guangzhou, Guangdong, China; 4 Shenzhen Key Laboratory of Spine Surgery, Department of Spine Surgery, Peking University Shenzhen Hospital, Shenzhen, Guangdong, China

**Keywords:** drug repurposing, ligamentum flavum hypertrophy, lipid accumulation, Mendelian randomization, placental alkaline phosphatase (ALPP), statins

## Abstract

**Background and objective:**

Lumbar spinal stenosis is a prevalent degenerative disorder driven primarily by ligamentum flavum hypertrophy. Emerging evidence links ectopic lipid accumulation to fibrotic remodeling and suggests a potential contribution of immune dysregulation. However, the molecular targets connecting lipid accumulation with ligamentum flavum fibrosis remain incompletely defined. We sought to evaluate the underlying mechanisms of lipid-lowering drug targets in lipid-associated ligamentum flavum fibrosis.

**Methods:**

We applied two-sample Mendelian randomization (MR), summary-data-based MR, and Bayesian colocalization to evaluate the associations of genetically proxied levels of 213 lipid-lowering drug target proteins with lumbar spinal stenosis risk. Candidate targets were evaluated *via* genetic immune-cell mediation analysis, molecular docking, molecular dynamics simulations, and experimental validation *in vitro* and *in vivo*.

**Results:**

MR identified three proteins increasing disease risk, with placental alkaline phosphatase (ALPP) consistently replicating across platforms (colocalization at rs12478529, PP.H4 = 0.9979). Genetically inferred mediation analysis suggested that selected immune-cell traits may partially mediate the association between ALPP and lumbar spinal stenosis. Molecular dynamics simulations and cellular thermal shift assays supported target engagement of atorvastatin and simvastatin with ALPP. In clinical specimens, ALPP was upregulated in hypertrophied tissues, co-localizing with fibrosis and lipid accumulation. *In vitro*, statins suppressed lipid accumulation and fibrotic markers; ALPP overexpression counteracted these protective effects. *In vivo*, systemic statin administration attenuated ligamentum flavum thickening, whereas local viral-mediated ALPP overexpression reversed this benefit.

**Conclusion:**

Our findings identify ALPP as a genetically supported candidate associated with LSS and link it to lipid accumulation and fibrosis. Computational and CETSA findings support possible target engagement of statins with ALPP and attenuate lipid accumulation and fibrosis, supporting ALPP as a potential mediator of their effects and statin repurposing for LSS.

## 1 Background

Lumbar spinal stenosis (LSS) is a debilitating condition characterized by narrowing of the spinal canal, leading to compression of the spinal cord or nerve roots (Yang et al., 2021). The pathological features of LSS mainly manifest as a thickening of the ligamentum flavum, herniated intervertebral discs, hypertrophy of the synovial joints, and the abnormal proliferation in bony or fibrous tissues, leading to a decrease in the volume of the spinal canal (Liu C. et al., 2022; Sun et al., 2021). This disease often results in pain, neurological dysfunctions, and a serious impact on the quality of life. The drugs currently used to treat LSS are non-steroidal anti-inflammatory drugs and other symptom-relieving drugs (Katz et al., 2022; Takeshita, 2023). Therefore, there is an urgent need to find drugs that target the pathogenesis of LSS.

Emerging evidence indicates that LF hypertrophy is not merely a mechanical wear-and-tear process, but rather a complex, localized fibrotic disorder involving metabolic and inflammatory alterations. Our prior work identified ACSM5 as a regulator of lipid deposition in this context, suggesting lipid modulation as a viable strategy to attenuate age-related spinal degeneration (Cao et al., 2023a). This “lipotoxic” microenvironment is believed to alter cellular epigenetics, thereby driving fibrosis and lipid metabolism disorders (Legchenko et al., 2018). Consequently, lipid-modulating pathways present a conceptually attractive avenue for intervening in LSS. Furthermore, lipotoxic microenvironments are known to trigger local immune responses, although the contribution of immune-cell alterations to LF fibrosis remains unclear (Shi et al., 2006).

Lipid-lowering drug target proteins have been shown to play important roles in various diseases (Peng et al., 2019; Kotyla and Kucharz, 2018). Drugs that decrease cholesterol primarily disrupt important proteins, enzymes, or receptors that are involved in the process of lipid metabolism. Furthermore, lipid-lowering agents like statins possess well-established immunomodulatory and anti-fibrotic properties independent of their systemic cholesterol-lowering effects (Jain and Ridker, 2005; Hu et al., 2026; Subramani et al., 2026). However, the precise causal relationship between specific lipid-lowering drug target proteins, lipid-associated fibrosis, and LSS has not been systematically elucidated.

In this study, we hypothesized that specific lipid-modulating proteins drive LF hypertrophy by promoting lipid accumulation and fibrotic remodeling, and that targeting these proteins could offer a novel therapeutic strategy. To test this, we integrated multi-platform Mendelian randomization (MR), genetic immune-cell mediation analysis, molecular docking, and molecular dynamics (MD) simulations with comprehensive *in vitro* and *in vivo* experimental models. We identified placental alkaline phosphatase (ALPP) as a genetically supported candidate to LSS, with genetic analyses suggesting a possible contribution of immune-cell traits. Furthermore, we provide evidence that statins engage ALPP and attenuate lipid accumulation, fibrosis, and LF hypertrophy, with ALPP contributing to these protective effects. These findings support ALPP as a candidate metabolic target for further evaluation of statin repurposing in LSS.

## 2 Materials and methods

### 2.1 Mendelian randomization and genetic association analyses

#### 2.1.1 Identification of lipid-lowering drug targets and pQTL data extraction

We collected target genes associated with lipid-lowering drugs (including statins, PCSK9 inhibitors, and others) by querying the DrugBank (https://go.drugbank.com/) and ChEMBL (https://www.ebi.ac.uk/) databases (Wishart et al., 2018; Mendez et al., 2019). The obtained protein UniProt IDs were subsequently converted to gene symbols utilizing the R package biomaRt (Version 2.54.1). After merging the datasets from both databases and removing duplicates, a total of 213 lipid-lowering drug-related target genes were identified (Supplementary Table 1).

Subsequently, protein quantitative trait loci (pQTL) data for these target genes were extracted from the deCODE database, which integrates large-scale plasma proteome and genetic data (Ferkingstad et al., 2021). The pQTL data comprised two distinct European-ancestry cohorts, defined as the Olink_UKB and SomaScan datasets, which served as our primary genetic instruments for identifying potential LSS drug targets. To ensure the analytical specificity of protein quantification given the homology among alkaline phosphatase family members, it is noteworthy that the Olink platform measures ALPP using Proximity Extension Assay (PEA) technology within the Target 96 Oncology III Panel. This approach utilizes a dual-antibody “gating” mechanism that effectively inhibits non-specific binding to paralogous proteins, such as ALPI, ALPL, and ALPPL2. Similarly, the SomaScan platform employs SOMAmer aptamers specifically designed for ALPP, the development of which incorporates a counter-selection step to eliminate cross-reactivity with homologous proteins. To ensure the robustness and specificity of the genetic instruments, strict criteria were applied to select cis-pQTLs (Yang et al., 2021): a genome-wide significance threshold of P < 5 × 10^−8^ (Liu C. et al., 2022); exclusion of single nucleotide polymorphisms (SNPs) located within the human major histocompatibility complex (MHC) region to prevent complex pleiotropy (Sun et al., 2021); selection of SNPs strictly positioned within a ±250 kb window around the encoding gene; and (Katz et al., 2022) linkage disequilibrium (LD) clumping with an r^2^ threshold of <0.2. All selected pQTL datasets were exclusively of European ancestry to mitigate bias from population stratification.

#### 2.1.2 Outcome data sets and other data sources

We obtained summary-level GWAS data for LSS outcomes from two independent European-ancestry datasets available in the MRC IEU OpenGWAS database. The FinnGen dataset (GWAS ID: finn-b-M13_SPINSTENOSIS), for which the phenotype was labeled as spinal stenosis, included 9,169 cases and 164,682 controls. The UK Biobank dataset (GWAS ID: ukb-d-M13_SPINSTENOSIS), which used the same phenotype label, included 1,910 cases and 359,284 controls. Both datasets included male and female participants of European ancestry, and the genomic coordinates were based on hg19/GRCh37. Standardized association summary statistics were retrieved as outcome data using the TwoSampleMR R package (version 0.6.6) (Hemani et al., 2018). Cases were defined according to the spinal stenosis phenotype codes provided in the respective databases. Because only publicly available summary-level GWAS data were used and no individual-level data were accessed, information on age distributions, detailed sex proportions, and other clinical and demographic characteristics was limited to that available in the public databases.

Other GWAS data: Based on prior literature, we selected three known risk factors for LSS—obesity, diabetes, and smoking—and retrieved their corresponding GWAS summary statistics (Shemesh et al., 2024; Ryu et al., 2025; Ryu et al., 2024). Each dataset was drawn from European ancestry populations to match the ancestry of our primary analysis and avoid bias from population stratification.

#### 2.1.3 Two-sample MR

We performed two-sample Mendelian randomization analyses using cis-pQTL data from the deCODE proteomic resource as exposures and GWAS summary statistics for LSS as outcomes (Ferkingstad et al., 2021). Exposure and outcome data were harmonized using the TwoSampleMR package (version 0.6.6), with effect alleles aligned across datasets. SNPs with incompatible alleles, unresolved strand orientation, or ambiguous palindromic alleles that could not be reliably aligned were excluded before analysis.

To assess instrument strength and reduce the risk of weak-instrument bias, we calculated the proportion of variance in the exposure explained by each SNP (*R* ^2^/PVE) and the corresponding F-statistic. For each SNP, *R* ^2^ was approximated as $R^{2}=2\times \text{MAF}\times \left(1-\text{MAF}\right)\times \beta^{2}$ , where MAF denotes the minor allele frequency and β denotes the SNP–protein effect estimate. The F-statistic for each SNP was calculated as follows (Equation 1):

$$F=\frac{N-k-1}{k}\times \frac{R^{2}}{1-R^{2}\;}$$ (1)

where N represents the exposure GWAS sample size. Only SNPs with MAF >0.01 and F-statistics >10 were retained for subsequent analyses. The F > 10 threshold was used to reduce, but not eliminate, the possibility of weak-instrument bias (Staiger and Stock, 1997; Burgess and Thompson, 2011).

For exposures instrumented by a single SNP, MR estimates were obtained using the Wald ratio method. Steiger directionality tests were performed to determine whether the instruments explained more variance in the exposure than in the outcome; the results were interpreted as supporting the specified exposure-to-outcome direction rather than verifying temporal or biological causality.

To account for multiple testing, Benjamini–Hochberg false discovery rate (BH-FDR) correction was applied to the MR P values for all tested proteins separately within each proteomic-platform-by-outcome analysis stratum. FDR-adjusted P values are reported as p.adjust. For the interpretation of cross-platform consistency, we required an association to remain statistically significant after BH-FDR correction in each platform-specific analysis and to show the same direction of effect across platforms. Nominally significant associations that did not survive BH-FDR correction were not considered evidence of cross-platform consistency.

The pQTL exposure data were obtained from the deCODE proteomic resource, whereas the LSS outcome data were obtained from FinnGen and UK Biobank. The FinnGen outcome cohort was considered largely independent of the exposure datasets. The degree of participant overlap between the pQTL exposure datasets and the UK Biobank LSS outcome dataset could not be fully determined from the available summary-level information. For analyses involving UK Biobank-based proteomic exposure data and UK Biobank LSS outcome data, partial sample overlap could not be completely excluded. Because only instruments with F-statistics >10 were retained, the susceptibility of the MR estimates to weak-instrument bias, including bias potentially amplified by sample overlap, was reduced. However, this restriction did not eliminate the potential influence of sample overlap, and this issue was therefore considered when interpreting the findings and acknowledged as a study limitation.

#### 2.1.4 Sensitivity analysis

Sensitivity analysis was performed using pleiotropy, heterogeneity, and one-by-one exclusion tests to guarantee the reliability of the results.

#### 2.1.5 Heterogeneity test

Heterogeneity between SNP estimates was assessed using the Cochran Q test. The analysis results were significantly heterogeneous if the Cochran’s Q test was statistically significant. The random-effects model of IVW was used to estimate the causal effect size for highly heterogeneous results. Because Cochran’s Q test can only evaluate for the presence or absence of heterogeneity, it cannot test the distribution of heterogeneity. Therefore, the I^2^ statistic was used to reflect the proportion of the heterogeneous part of the instrumental variable in the total variation: when I^2^ ≤ 0, it was set to 0, indicating that no heterogeneity was observed. Furthermore, I^2^ = 0∼25%, mild heterogeneity; I^2^ = 25∼50%, moderate heterogeneity; I2>50%, high heterogeneity. The calculation formula is as follows (Equation 2)

$$I^{2}=\frac{Q-df}{Q}\times 100\%$$ (2)

#### 2.1.6 Pleiotropy test

The pleiotropy of the instrumental variables was examined using the MR-Egger method. Significant horizontal pleiotropy of genetic variation was indicated if the P-value of the MR-Egger’s intercept was <0.05.

#### 2.1.7 Leave-one-out test

Individual SNPs were eliminated one at a time from the MR results for the remaining instrumental variables to ascertain whether the SNP had an impact on the association between the target protein and LSS. The MR effect estimates may have been sensitive to that SNP if there was a substantial difference between them and the total effect estimates after an instrumental variable was eliminated.

#### 2.1.8 SMR analysis

SMR (summary-data-based MR) determines pleiotropic relationships between base protein expression levels and complex traits of interest using GWAS summary data and expression quantitative trait locus research (Zhu et al., 2016). The colocalization signal is examined for potential horizontal pleiotropy using the HEIDI (heterogeneity in dependent instruments) test. We downloaded SMR Linux version 1.3.1 from the SMR website (https://yanglab.westlake.edu.cn/software/smr) using the default settings for SMR analysis.

#### 2.1.9 Colocalization analysis

We performed colocalization analysis using the COLOC program for genes with substantial MR relationships (Giambartolomei et al., 2014). A Bayesian technique was employed to ascertain whether shared causal variations at particular loci, rather than linkage disequilibrium, caused a connection between protein expression and LSS. Five assumptions underlie the colocalization analysis: H0 and SNPs in the colocalization zone are unrelated to either characteristic, H1 SNPs within the colocalization region were associated with the first trait but not with the second trait, H2 and SNPs within the colocalization region were associated with the second trait but not with the first trait, H3 SNPs within the colocalization region were associated with both traits, but not with the same locus, and H4 SNPs within the colocalization region were associated with both traits and were located at the same locus. We considered H4 ≥ 0.8 as a high level of colocalization and 0.5 ≤ H4≤ 0.8 as a moderate level of colocalization (Zhou et al., 2021).

#### 2.1.10 Phenome-wide association analysis (PheWAS)

To evaluate the pleiotropic effects and potential adverse reactions of the identified target protein (ALPP) as therapeutic targets, we conducted comprehensive analyses on the filtered protein candidates using two publicly available databases: the AstraZeneca PheWAS Portal (https://azphewas.com/) and the PheWeb Database (https://pheweb.org/) (Wang et al., 2021; Gagliano Taliun et al., 2020). The studies hosted on these platforms cover roughly 15,500 binary and 1,500 continuous phenotypes, all derived from exome sequences of the UK Biobank cohort.

#### 2.1.11 Multivariate MR analysis and mediating effect estimation

Multivariable MR (MVMR), an extension of standard MR, was utilized to evaluate the direct effects of the exposures on the outcome. To determine the direct impact of the target proteins on spinal stenosis, MVMR analysis was conducted by incorporating the selected exposure proteins alongside established risk factors for spinal stenosis.

Furthermore, to explore the indirect effects, we investigated whether the target proteins influence spinal stenosis through immune cell-mediated pathways. GWAS summary data for an established list of immune cell traits were obtained by reviewing the literature (Supplementary Table 2), with only samples of European ancestry retained to ensure population consistency.

The mediating pathway was quantified through a two-step MR approach. The effect sizes and standard errors of the mediating (indirect) effects were calculated using the following equations:

$$\beta_{M}={\;\beta }_{A}\times \beta_{B}$$

$${\text{SE}}_{M}=\sqrt{{\left(\beta_{A}\times {\text{SE}}_{B}\right)}^{2}+{\left(\beta_{B}\times {\text{SE}}_{A}\right)}^{2}-{{\text{SE}}_{A}}^{2}\times {\;{\text{SE}}_{B}}^{2}}$$

Where SE_M_ stands for the standard error of the mediating effect, SE_A_ is the standard error of the MR analysis of the exposed protein on immune cells, SE_B_ is the standard error of the MR analysis of immune cells on spinal stenosis, β_M_ is the effect value of mediating effect, β_A_ is the MR effect value of exposed protein on immune cells, and β_B_ is the direct effect value of immune cells on spinal stenosis.

Combined with the causal stepwise regression method, if β_A_ and β_B_ are both significant, the indirect effect is significant. If β_A_ and β_B_ were not significant, the Sobel test was used to determine if β_M_ was significant. If β_M_ was significant, the indirect effect was also significant. Based on the significant indirect effect, if β_M_ was different from the MR effect value, β_C′_, of the exposed protein on spinal stenosis in MVMR, the ratio of the mediating effect of the exposed protein on spinal stenosis through immune cells was calculated as |β_M_/β_C′_|×100%. If β_M_ was the same as the MR effect value β_C′_ of the exposed proteins on spinal stenosis in MVMR, the proportion of the mediating effect of the exposed proteins on spinal stenosis through immune cells in the total effect was calculated as (β_M_/β_C_) ×100%, where β_C_ is the effect value of the exposed proteins on spinal stenosis in the univariate MR.

### 2.2 Molecular docking and molecular dynamics (MD) simulations

#### 2.2.1 Molecular docking simulations

To explore the therapeutic potential of statin repurposing against the ALPP target, blind protein-ligand docking was executed using the CB-Dock2 server, which integrates AutoDock Vina (Liu Y. et al., 2022; Trott and Olson, 2010). The 3D structure of ALPP was obtained from the PDB database, and the structures of six statins (lovastatin, simvastatin, pravastatin, atorvastatin, rosuvastatin, and pitavastatin) were retrieved from the PubChem database (Berman et al., 2000; Kim et al., 2021). Protein-ligand docking was performed using the CB-Dock2 server (integrating AutoDock Vina). Binding affinities (Vina scores) and intermolecular interactions (e.g., hydrogen bonds, hydrophobic interactions) were systematically analyzed.

#### 2.2.2 MD simulations and binding free energy calculations

Protein and compound 3D structures (PDB format) were acquired. Following structural optimization of the compounds, their binding sites were determined utilizing AI predictions, databases, and relevant literature. We used molecular docking and the resulting complexes provided the initial coordinates for molecular dynamics (MD) simulations.

All-atom explicit solvent systems were constructed using GROMACS 2024.05 (Pronk et al., 2013). The protein was parameterized with the AMBER99SB-ildn force field and solvated in a TIP3P water model (Lindorff-Larsen et al., 2010). Ligand topologies were assigned utilizing the General AMBER Force Field (GAFF). For the small molecules, Restrained Electrostatic Potential 2 (RESP2) charges were calculated via Multiwfn following ORCA geometry optimization, and force field parameters were generated via Sobtop (Lu and Chen, 2012).

Following solvation, the systems underwent steepest-descent energy minimization (50,000 steps) to eliminate spatial conflicts. Subsequently, sequential equilibrations were performed under the NVT and NPT (1 bar) ensembles for 100 ps (50,000 steps) each. System temperature and pressure were maintained at 310 K and 1 bar using the V-rescale and Berendsen methods, respectively. Non-bonded interactions were calculated using a Verlet buffer, updating the neighbor list every 10 steps with a 1.2-nm cutoff distance. Finally, completely unrestrained production MD simulations were executed for 100 ns with a 2-fs time step, saving trajectories every 10 ps. Protein-ligand binding free energies and free energy decompositions were quantified using gmx_MMPBSA (Valdés-Tresanco et al., 2021).

### 2.3 Clinical specimen assessment

Human ligamentum flavum (LF) specimens at the L4/5 level were collected from 24 patients who underwent lumbar surgery at Zhujiang Hospital following approval by the Institutional Research Ethics Committee (No. 2021-ky-122-01) and provision of written informed consent. The hypertrophied LF group included 12 patients with lumbar spinal stenosis (LSS) and an LF thickness >4 mm, whereas the reference group included 12 patients with lumbar disc herniation (LDH), without radiographic LF hypertrophy, and an LF thickness ≤ 4 mm. Age, sex, body mass index (BMI), LF thickness, spinal canal area, and statin use were obtained from the clinical records and are summarized in Supplementary Table 23. Because LF specimens cannot ethically be collected from healthy individuals, non-hypertrophied LF tissues from patients undergoing surgery for LDH were used as surgical reference tissues rather than as healthy controls. Specimens were fixed in 4% PFA and then were sent to be paraffin-embedded or cryoprotected. Histological architecture, fibrosis and lipid accumulation were assessed using H&E, Safranin O-Fast Green, Masson’s trichrome, Elastic Van Gieson, Picrosirius Red and Oil Red O staining. For detecting ALPP expression, immunohistochemistry (IHC) was performed using anti-ALPP antibodies (1:100, Proteintech) with DAB visualization. Finally, stained slides were scanned with a slide scanner (HISTECH) for image acquisition. For each sample, the percentage of DAB-positive area relative to the total tissue area was quantified in three randomly selected, non-overlapping fields in ImageJ, and the values were averaged.

### 2.4 *In Vitro* pathological modeling and target validation

#### 2.4.1 Cell culture and treatment

Primary ligamentum flavum (LF) cells were isolated from surgical specimens obtained from 5 independent donors (2 males and three females; age range, 60–63 years). Cells derived from different donors were cultured and analyzed separately. Cells at passages 3-5 were used for all experiments. To simulate the lipotoxic microenvironment and induce intracellular lipid accumulation, LF cells were treated with an FFA mixture at a total final concentration of 200 μM, consisting of 133.3 μM oleic acid (OA; Sigma-Aldrich) and 66.7 μM palmitic acid (PA; Sigma-Aldrich) at an OA:PA molar ratio of 2:1. The FFA mixture was prepared according to a previously described protocol (Fang et al., 2025) and diluted in phosphate-buffered saline (PBS). Control cells received an equivalent volume of PBS. Cells were exposed to the FFA mixture for 24h. Safe working concentrations for the FFA and statins (Selleck, Shanghai, China) were determined via Cell Counting Kit-8 (CCK-8, Beyotime, China) viability assays. As a result, 200 µM FFA concentration was ultimately established for pathological modeling. For gene overexpression studies, a plasmid overexpressing human ALPP (*OE-ALPP*) and its negative control (OE-NC) were constructed by GenePharma (Suzhou, China). Transfections were performed using Lipofectamine 3,000 (Thermo Fisher Scientific) according to the manufacturer’s protocol. Briefly, LF cells were seeded in 6-well plates, and FFA along with statin treatments were added 24 h after transfection with *OE-ALPP*.

For statin treatments, atorvastatin (10 μM), simvastatin (10 μM), pravastatin (10 μM), and pitavastatin (1 μM) were prepared in PBS. Vehicle-control cells received the same concentration of the vehicle without statins.

#### 2.4.2 Oil Red O staining and Nile Red fluorescent staining

Intracellular lipid droplet accumulation was quantified using Oil Red O and Nile Red fluorescent staining assays (Solarbio, China) following the manufacturer’s standard protocols. Briefly, post-treatment LF cells were washed with PBS and fixed in 4% paraformaldehyde (PFA) for 20 min. For Oil Red O staining, fixed cells were incubated with a freshly filtered Oil Red O working solution for 15 min at room temperature, washed with PBS, and visualized under a bright-field microscope. For Nile Red fluorescent staining, cells were incubated with the Nile Red probe for 15 min in the dark, followed by nuclear counterstaining with DAPI. Images were captured using a fluorescence microscope, and the lipid droplet areas or fluorescence intensities were quantified using ImageJ software.

#### 2.4.3 Western blotting

Western blotting was conducted as previously described (Cheng et al., 2026). Briefly, total proteins from LF cells or tissues were extracted using RIPA lysis buffer containing protease and phosphatase inhibitors (Beyotime, China). Equal amounts of protein lysates (30 μg) were separated by SDS-PAGE gel electrophoresis and then transferred onto 0.45 μm polyvinylidene fluoride (PVDF) membranes (Millipore, USA). The membranes were blocked with QuickBlockTM blocking buffer (Beyotime, China) for 0.5 h at room temperature, followed by incubation with primary antibodies overnight at 4 °C. After washing the membranes three times with TBST, they were incubated with diluted HRP-conjugated secondary antibodies for 2 h at room temperature. Finally, the protein bands were imaged by a qTouch Western blot imager (RWD, China) using an ECL Kit (Vazyme, Nanjing) and quantified with ImageJ software. Detailed antibody information is provided in Supplementary Table 3.

#### 2.4.4 Real-time quantitative PCR (RT-qPCR)

RT-qPCR was performed as previously described (Cheng et al., 2026). Briefly, total RNA was extracted from the LF cells using TRIzol reagent (Invitrogen, Carlsbad, CA, USA) and subsequently reverse-transcribed into cDNA using the PrimeScript™ RT reagent Kit with gDNA Eraser (Takara, Dalian, China). RT-qPCR amplification was performed on a LightCycler® 96 RT-qPCR instrument (Roche) using SYBR Green qPCR Mix (Beyotime, China). Then, the relative mRNA expression levels of target genes were evaluated utilizing the 2^−ΔΔCT^ method. The specific primer sequences used in this study are listed in Supplementary Table 4.

#### 2.4.5 Cellular thermal shift assay (CETSA)

CETSA was conducted to evaluate intracellular target engagement (Martinez Molina et al., 2013), following specific protocols as previously described (Cheng et al., 2026). Briefly, LF cells treated with statins or vehicle were subjected to a temperature gradient (37 °C–62 °C) for 3 min by a thermal cycler (Eppendorf, Germany), followed by centrifugation. The soluble protein fractions were subsequently analyzed by Western blotting to assess ALPP thermal stabilization.

#### 2.4.6 Immunofluorescence

Immunofluorescence was performed to evaluate specific target protein expression in LF cells and mouse LF tissues. Briefly, cells cultured on glass coverslips were fixed with 4% paraformaldehyde for 15 min and permeabilized with 0.2% Triton X-100 for 5 min. To achieve high-sensitivity protein detection, a TSA (Tyramide Signal Amplification)-based detection kit (Proteintech, China) was utilized. Non-specific binding was blocked using the provided Blocking/Dilution Buffer for 30 min at room temperature. Cells were then incubated with the primary antibody overnight at 4 °C. After washing three times with PBS, cells were incubated with the Multi-rAb™ Polymer HRP-Goat Anti-Rabbit Secondary Antibody for 30min at room temperature. Signal amplification was subsequently achieved by applying the CoraLite®Plus 488-Tyramide and CoraLite®Plus 647-Tyramide working solution (diluted 1:50) for 10 min in the dark. Nuclei were stained with Hoechst 33342 and observed under a fluorescence microscope (Nikon Ti2-E).

### 2.5 *In Vivo* animal models

All experiments and surgical procedures were approved by the Animal Ethics Committee of Zhujiang Hospital, Southern Medical University (LAEC-2023-121). Eight-week-old male C57 mice were purchased from Guangdong Medical Laboratory Animal Center. A mouse model of LF hypertrophy was established according to previously described protocols utilizing the water-aversion-induced bipedal standing behavior of mice (Duan et al., 2022).

To explicitly define the experimental groups: Sham represents non-loaded controls (housed in identical cylindrical containers without water at the bottom, thereby avoiding bipedal standing); Model represents mice subjected to bipedal-loading to induce LF hypertrophy; and AAV-NC represents the empty-vector viral control. To evaluate whether ALPP contributes to the effects of statins on LF hypertrophy *in vivo*, the modeled mice were randomly assigned into the following groups: Sham; Model; Model + atorvastatin; Model + simvastatin; Model + simvastatin + AAV-NC; Model + atorvastatin + AAV-NC; Model + atorvastatin + *AAV*-*ALPP* and Model + simvastatin + *AAV-ALPP* (n = 5 per group). Adeno-associated virus (AAV) for ALPP overexpression in mice was purchased from GenePharma (Suzhou, China). AAV-NC had the same viral backbone as *AAV-ALPP* but did not contain the ALPP coding sequence. The same viral titer, injection volume, and surgical procedure were used for the AAV-NC and *AAV-ALPP* groups. The total experimental timeline spanned 16 weeks. At the end of the sixth week of the modeling period, mice designated for AAV injection were anesthetized with isoflurane (2% for induction, 1.5% for maintenance). The mice were placed in the prone position on a heating pad maintained at 37 °C to prevent hypothermia. After shaving and disinfecting the lumbar dorsal skin with 75% ethanol and povidone-iodine, a longitudinal midline skin incision (∼1 cm) was made over the lumbar spine centered at the L5/L6 level, which was identified using the iliac crest as an anatomical landmark. The dorsal paravertebral muscles were carefully bluntly dissected from the spinous processes and laminae bilaterally to expose the L5/L6 interlaminar space and the LF. Subsequently, precisely 3 μL of the designated viral vector was locally injected into the bilateral L5/L6 LF. The surgical wound was irrigated with sterile saline, and the fascia and skin were closed in layers with 5-0 absorbable sutures. Postoperative analgesia was provided by subcutaneous injection of buprenorphine (0.05 mg/kg) every 12 h for 3 consecutive days. Mice were monitored on a warming pad until full recovery from anesthesia and subsequently returned to their respective housing containers to continue the bipedal standing protocol. Statin doses were determined based on previously published murine studies. Specifically, mice in the statins group received simvastatin (5 mg/kg/day, Selleck) or atorvastatin (10 mg/kg/day, Selleck) dissolved in PBS by daily oral gavage (Zakyrjanova et al., 2025; Panajatovic et al., 2021). The corresponding Sham and model groups received an equal volume of vehicle. These daily statin or vehicle treatments were initiated immediately after the AAV injection and were administered concurrently with the bipedal-standing protocol for a duration of 10 weeks. At the end of this 16-week total experimental period, the LF tissues of the mice were collected.

### 2.6 Statistical methods

Given the integration of large-scale genetic epidemiology and biological experiments, statistical analyses were conducted utilizing distinct analytical platforms. For MR, SMR, colocalization, and related bioinformatic evaluations, all data processing and statistical calculations were executed using R software (version 4.2.2; https://www.r-project.org/). For the quantitative evaluation of clinical specimens, *in vitro* cellular assays, and *in vivo* animal models, all data were presented as the means ± standard deviation (SD) based on at least three independent experiments. These statistical analyses were performed using GraphPad Prism software (GraphPad Software, USA). Statistical significance between two independent experimental groups was analyzed using the two-tailed unpaired Student’s t-test. Differences among three or more groups were evaluated using a one-way analysis of variance (ANOVA) followed by Tukey’s *post hoc* test for multiple comparisons. A p-value <0.05 was considered to indicate a statistically significant difference unless otherwise specified. For cellular experiments, biological replicates were defined as cells obtained from independent donors. Thus, the reported n represents the number of independent biological donors. For the *in vivo* histological analyses, tissue sections and images were anonymized using coded identifiers. LF cross-sectional area and Oil Red O-positive area were quantified by two investigators blinded to the treatment allocation.

## 3 Results

### 3.1 Technical Roadmap

A schematic representation detailing the comprehensive research framework and procedural workflow adopted in this study is shown in Figure 1. Furthermore, a detailed step-by-step flowchart specifically delineating the data sources, instrumental variable selection criteria, and bioinformatic pipelines for the Mendelian randomization analyses is provided in Supplementary Figure 1.

**FIGURE 1:**
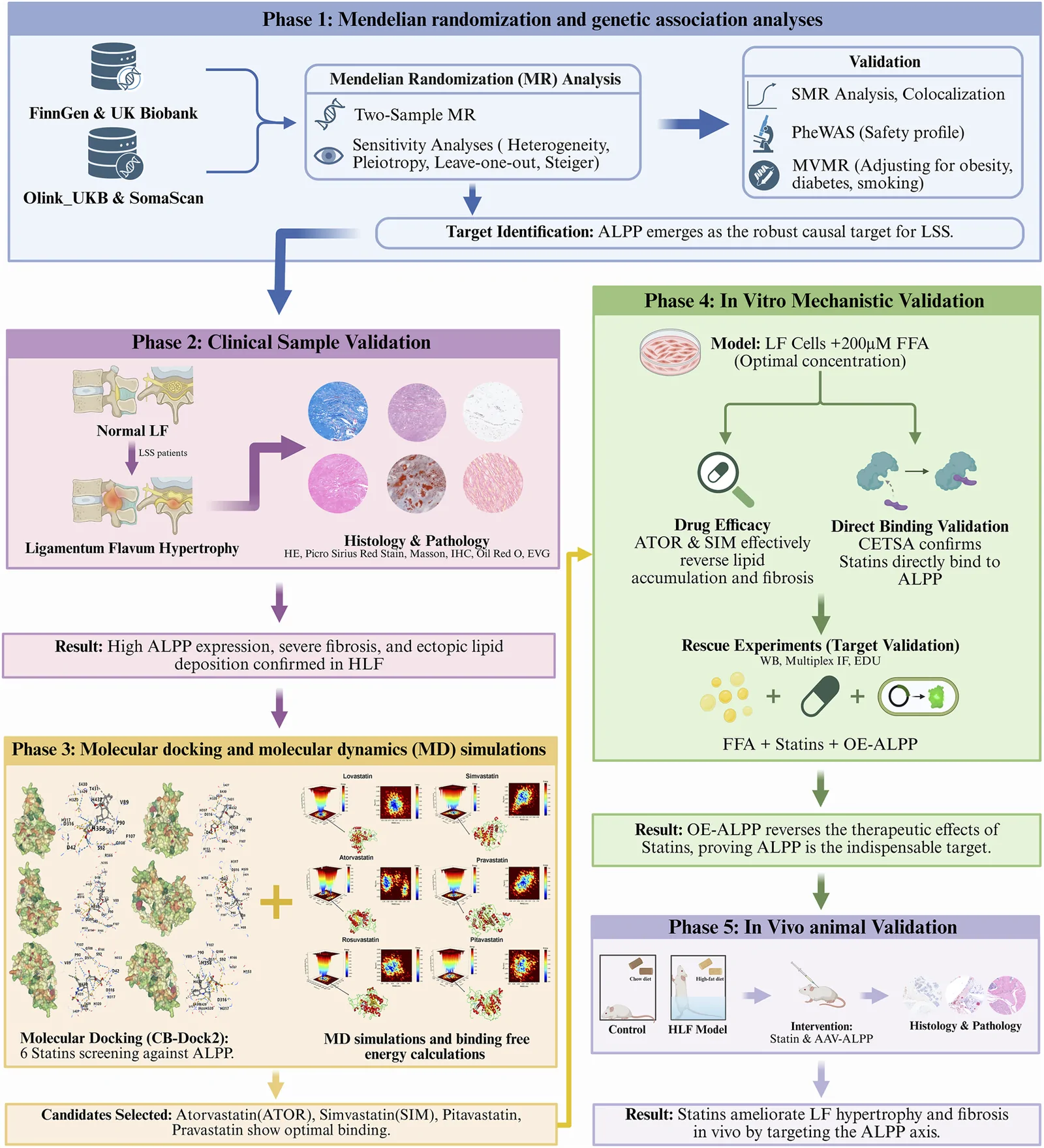
Overview of the design and methods used in this Mendelian randomization study. MR, Mendelian Randomization; SMR, Summary-data-based Mendelian Randomization; pQTL, protein Quantitative Trait Locus; HEIDI, Heterogeneity in Dependent Instruments.

### 3.2 MR analysis and sensitivity analysis of target proteins

We initiated the analysis by retrieving pQTL datasets for lipid-lowering drug target proteins from two independent platforms within the deCODE database: Olink UKB and SomaScan. Following established criteria, we screened the data and identified 30 and 31 target proteins with eligible cis-pQTL (Supplementary Tables 5, 6) corresponding to the target proteins. Two-sample Mendelian randomization (MR) analysis identified three proteins (BMP6, ALPP, and EPHB4) significantly associated with an increased risk of LSS (Table 1). Of all proteins tested, only ALPP showed a consistent, risk-increasing genetically predicted association on LSS—and it did so across both proteomic platforms (Olink and SomaScan) and in both independent cohorts (UK Biobank and FinnGen) (Table 2; Figure 2).

**TABLE 1:** Mendelian Randomization Analysis of the Causal Effect of deCODE’s olink_ukb-Sourced Proteins on the Development of Spinal Stenosis.

Exposure	Outcome	nsnp	Beta	SE	OR (95% CI)	p.adjust	Method
BMP6	finn-b-M13_SPINSTENOSIS	23	0.451174	0.084684	1.570155	5.97E-06	Inverse variance weighted
ALPP	ukb-d-M13_SPINSTENOSIS	70	0.000643	0.000159	1.000643	0.003258	Inverse variance weighted
EPHB4	ukb-d-M13_SPINSTENOSIS	23	0.002036	0.000576	1.002038	0.024338	Inverse variance weighted
DUSP13	finn-b-M13_SPINSTENOSIS	26	−0.14708	0.0506	0.863228	0.219191	Inverse variance weighted
EGFR	finn-b-M13_SPINSTENOSIS	39	0.115568	0.043473	1.122511	0.471097	Inverse variance weighted
INPP5D	finn-b-M13_SPINSTENOSIS	17	0.188933	0.073989	1.20796	0.639817	Inverse variance weighted
ACP1	finn-b-M13_SPINSTENOSIS	35	−0.06586	0.02643	0.936262	0.762456	Inverse variance weighted
ACP1	ukb-d-M13_SPINSTENOSIS	33	0.000716	0.000288	1.000716	0.768498	Inverse variance weighted
PCSK9	ukb-d-M13_SPINSTENOSIS	30	0.000786	0.00033	1.000786	1	Inverse variance weighted
DPP4	ukb-d-M13_SPINSTENOSIS	24	0.000959	0.000463	1.00096	1	Inverse variance weighted

SNP, single nucleotide polymorphism; OR, odds ratio; CI, confidence interval; SE, standard error.

**TABLE 2:** Mendelian Randomization Causal Effect Estimates of Somascan-Derived Proteins from deCODE on the Pathogenesis of Spinal Stenosis.

Exposure	Outcome	nsnp	Beta	SE	OR (95% CI)	p.adjust	Method
ALPP	ukb-d-M13_SPINSTENOSIS	54	0.001053	0.000265	1.001054	0.004174	Inverse variance weighted
NT5C2	finn-b-M13_SPINSTENOSIS	7	0.296636	0.09216	1.345326	0.078553	Inverse variance weighted
NT5C	ukb-d-M13_SPINSTENOSIS	20	−0.0007	0.000301	0.999301	1	Inverse variance weighted
ACP1	ukb-d-M13_SPINSTENOSIS	73	−0.00027	0.000115	0.999731	1	Inverse variance weighted
ALPP	finn-b-M13_SPINSTENOSIS	50	−0.05486	0.025313	0.94662	1	Inverse variance weighted

SNP, single nucleotide polymorphism; OR, odds ratio; CI, confidence interval; SE, standard error.

The two ALPP, estimates derived from the SomaScan dataset were obtained from separately specified analytical records and are therefore reported with explicit outcome-cohort and instrument-set labels. The positively signed association remained statistically significant after BH-FDR, correction (β = 0.001053, adjusted P = 0.004174). The negatively signed estimate did not survive multiple-testing correction (β = −0.05486, adjusted P = 1.000) and should not be interpreted as evidence of a protective effect. Cross-platform consistency was assessed only for associations that remained significant after BH-FDR, correction and showed concordant directions of effect. nsnp denotes the number of SNP, instruments included in each MR, analysis.

**FIGURE 2:**
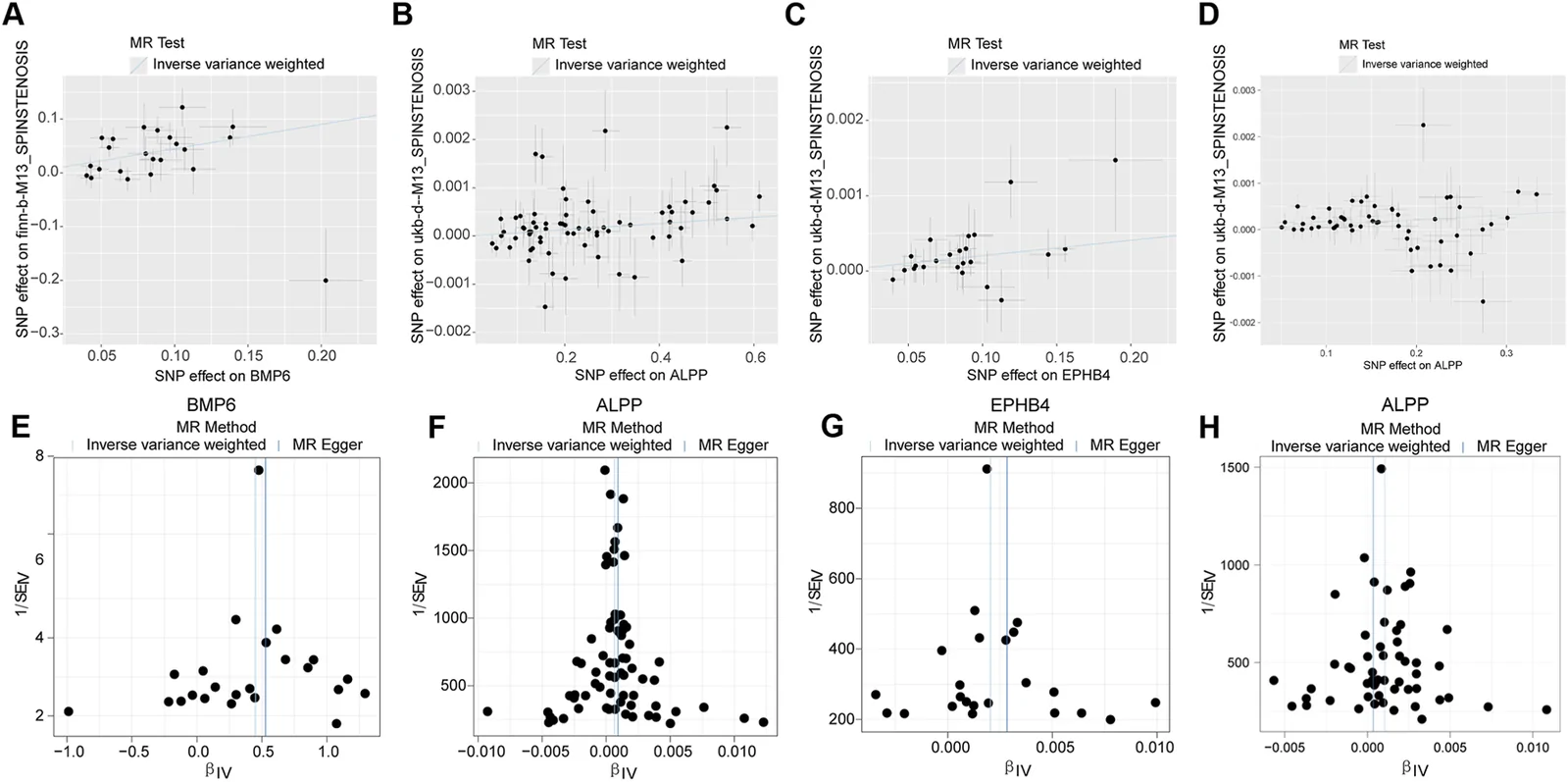
Mendelian randomization (MR) analyses evaluating genetically predicted associations between circulating proteins and lumbar spinal stenosis. **(A)** MR scatter plot evaluating the causal effect of BMP6 on the risk of spinal stenosis. The solid line indicates the inverse variance weighted (IVW) estimate. **(B)** MR scatter plot evaluating the causal effect of ALPP on spinal stenosis. **(C)** MR scatter plot for EPHB4. **(D)** MR scatter plot for ALPP. **(E)** Funnel plot assessing heterogeneity and directional pleiotropy in the MR analysis of BMP6. Vertical lines indicate the IVW and MR Egger estimates. **(F)** Funnel plot for ALPP. **(G)** Funnel plot for EPHB4. **(H)** Funnel plot for ALPP.

We then applied Cochran’s Q tests to evaluate heterogeneity in the MR estimates. For the Olink UKB-derived proteins, neither ALPP nor EPHB4 displayed heterogeneity (all p > 0.05; Supplementary Table 7). The same held for ALPP derived from the SomaScan platform (p > 0.05; Supplementary Table 8).

To assess the robustness of these MR estimates, we carried out a series of sensitivity analyses. First, MR-Egger intercept tests did not provide statistical evidence of directional horizontal pleiotropy across both the Olink- and SomaScan-derived datasets, with all intercepts approximating zero (p > 0.05) (Supplementary Tables 9, 10). Furthermore, leave-one-out analyses suggested that the estimates were not driven by any single variant, confirming that the overall causal estimates were not disproportionately driven by any single genetic variant (Figures 2E–H). Crucially, to mitigate the risk of reverse causation, Steiger directionality tests unequivocally validated the presumed forward sequence—demonstrating that protein alterations significantly precede LSS pathogenesis (p < 0.05) (Supplementary Tables 11, 12).

### 3.3 SMR analysis and colocalization analysis

Subsequently, we identified the key protein ALPP through the intersection of the MR and SMR results for downstream analyses. Specifically, SMR analyses confirmed the associations, and the HEIDI test results (P_HEIDI_ > 0.05) indicated that these signals were not driven by linkage disequilibrium (Supplementary Tables 13, 14). We next performed Bayesian colocalization analyses on the top independent SNPs at the locus encoding the ALPP protein (Table 3). For the lead SNP derived from the SomaScan platform (rs12478529), the colocalization was highly robust (PP.H4 = 0.9979). This result provides genetic support for a shared association signal at this locus: a PP.H4 value approaching unity at rs12478529 supports the possibility that the protein and outcome associations share a causal variant within this genomic region, indicating that the pathogenic signal at the locus encoding ALPP is tightly localized. Consequently, this integrated evidence supports prioritizing ALPP as a candidate for further functional and therapeutic investigation.

**TABLE 3:** Results of colocalization analysis between protein and spinal stenosis.

Exposure	topsnp	PP.H4.abf
ALPP (SomaScan – sourced)	rs12478529	0.997916

### 3.4 Phenome-wide association analysis

We used whole-phenome MR to probe whether targeting ALPP might carry unintended risks. Across all traits tested, no association reached genome-wide significance (every p-value exceeded 5 × 10^−8^; Supplementary Tables 15, 16; Figure 3). These findings indicate a highly specific target profile, highlighting a minimal risk of off-target adverse effects and further corroborating the absence of horizontal pleiotropy at this locus.

**FIGURE 3:**
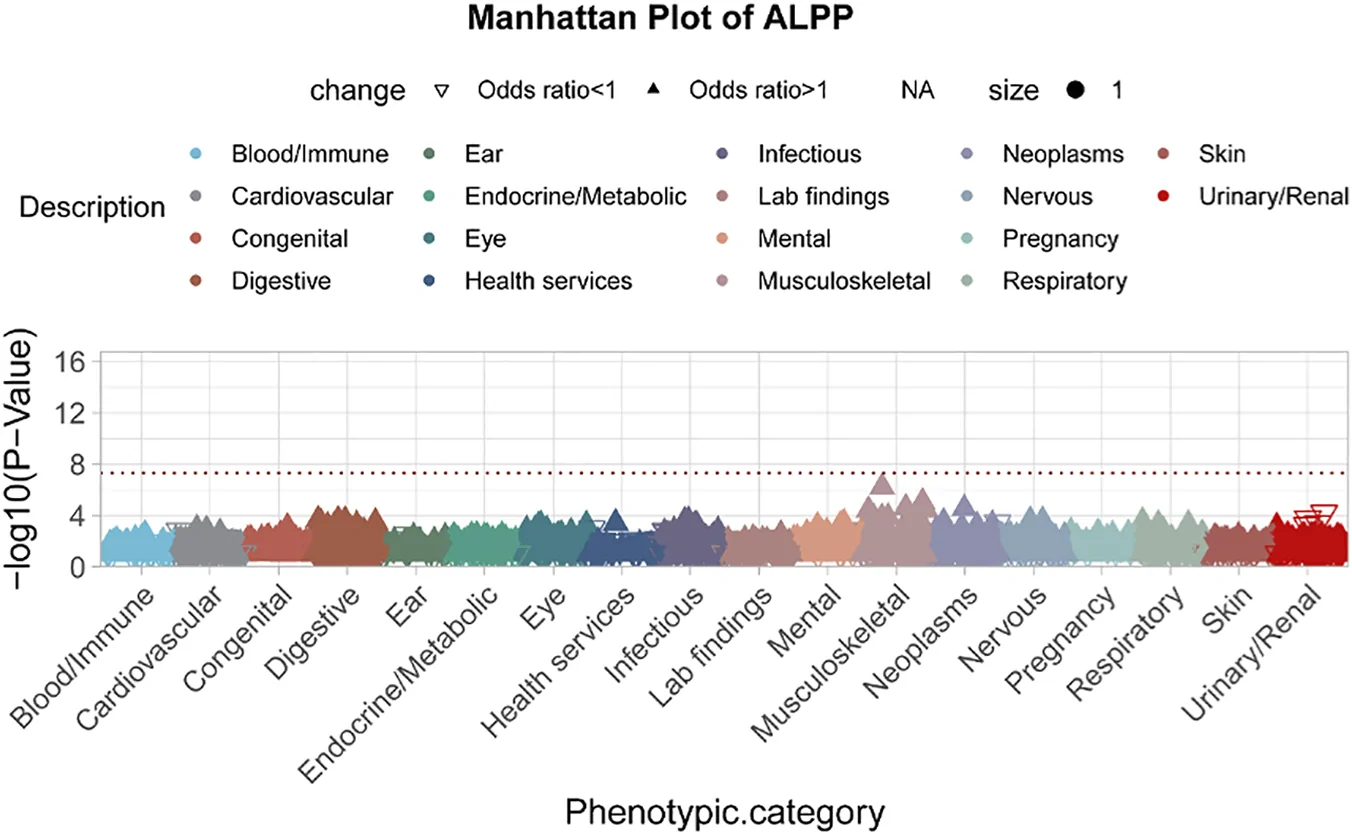
Manhattan plot of genome-wide association analysis for ALPP protein. The Manhattan plot illustrates genome-wide association signals for ALPP protein across all analyzed loci.

### 3.5 Multivariate MR analysis

We used multivariate MR (MVMR) to test whether ALPP affects LSS directly, after adjusting for three established risk factors: obesity, diabetes, and smoking. Across both the Olink_UKB (Table 4) and SomaScan (Table 5) datasets, the causal effect of ALPP on LSS held up—it was not attenuated by conditioning on these confounders. ALPP thus appears to act on LSS through pathways independent of metabolic or smoking-related mechanisms, which supports treating it as a direct therapeutic target. This reasoning informed our subsequent drug screening analyses.

**TABLE 4:** Multivariable MR analysis of ALPP on LSS risk using Olink UKB data.

Model	Exposure	Outcome	Standard error	P value
Model1	ALPP	SPINSTENOSIS	0.000129	0.0001
	Smoking behavior (cigarettes smoked per day)	SPINSTENOSIS	0.000439	0.283082
Model2	ALPP	SPINSTENOSIS	0.000127	6.23E-05
	Obesity	SPINSTENOSIS	0.000404	0.047424
Model3	ALPP	SPINSTENOSIS	0.00013	0.000243
	Diabetes diagnosed by doctor	SPINSTENOSIS	0.004018	0.166271

**TABLE 5:** Multivariable MR analysis of ALPP on LSS risk using SomaScan data.

Model	Exposure	Outcome	Standard error	P value
Model1	ALPP	SPINSTENOSIS	0.000216	3.36E-08
	Smoking behavior (cigarettes smoked per day)	SPINSTENOSIS	0.000645	0.703354
Model2	ALPP	SPINSTENOSIS	0.000209	2.33E-08
	Obesity	SPINSTENOSIS	0.000426	0.370684
Model3	ALPP	SPINSTENOSIS	0.000225	1.25E-05
	Diabetes diagnosed by doctor	SPINSTENOSIS	0.00405	0.08767

MR, mendelian randomization.

### 3.6 Mediation effect analysis

For mediation effect analysis, we first used the IVW model. The IVW model results showed that the two sources of ALPP (Olink_UKB source [Supplementary Table 17] and Somascan source [Supplementary Table 18]) had a significant causal relationship with immune cells. We then determined whether the causal direction of the two sources of ALPP to immune cells was corrected by the Steiger directivity test, which calculated the variance explanation rate (r^2^) of the SNPs for exposure and outcome risk factors, respectively. The results showed that the variance explanation rate (r^2^) of the SNPs for the indicators selected for the protein ALPP from the two sources was greater than that for the risk factors of the outcome, and the direction was TRUE and P < 0.05, which was significantly correct. We then performed multivariate MR analysis with the two sources of protein ALPP (OLINK_UKB [Supplementary Table 19] and SOMascan [Supplementary Table 20]) and immune cells and evaluated the mediating effect of the model, wherein the mediating factors in the multivariate MR analysis had a significant causal relationship with the outcome. In the multivariate model, immune cells had a significant effect on spinal stenosis (P < 0.05). Therefore, selected immune-cell traits showed evidence of partial genetic mediation in the association between ALPP and spinal stenosis.

According to the univariable and multivariable MR results, several immune-cell traits showed statistically supported indirect effects in the association between genetically proxied ALPP levels and spinal stenosis. For Olink_UKB-derived ALPP, ebi-a-GCST90001988 and ebi-a-GCST90001991 showed indirect effects consistent with partial mediation (Supplementary Table 21). Similar evidence was observed for SomaScan-derived ALPP and ebi-a-GCST90001532, ebi-a-GCST90001912, ebi-a-GCST90001944, ebi-a-GCST90001945, and ebi-a-GCST90002007 (Supplementary Table 22), whereas most other models showed masking effects. Collectively, these analyses suggest that selected immune-cell traits may partially contribute to the ALPP–LSS association; however, direct immune-cell involvement remains to be experimentally validated.

### 3.7 Molecular docking

Using CB-Dock2, we docked the active ingredients of six statins against ALPP (Figure 4). All six statins exhibited favorable binding profiles, with Vina scores ranging from −6.8 to −7.5 kcal/mol. Notably, pitavastatin yielded the most favorable docking score (Vina score = −7.5 kcal/mol; Figure 4F), followed closely by atorvastatin (−7.2 kcal/mol; Figure 4D) and simvastatin (−7.1 kcal/mol; Figure 4B). Lovastatin, pravastatin, and rosuvastatin also displayed moderate-to-strong interactions (−6.8 to −6.9 kcal/mol; Figures 4A,C,E).

**FIGURE 4:**
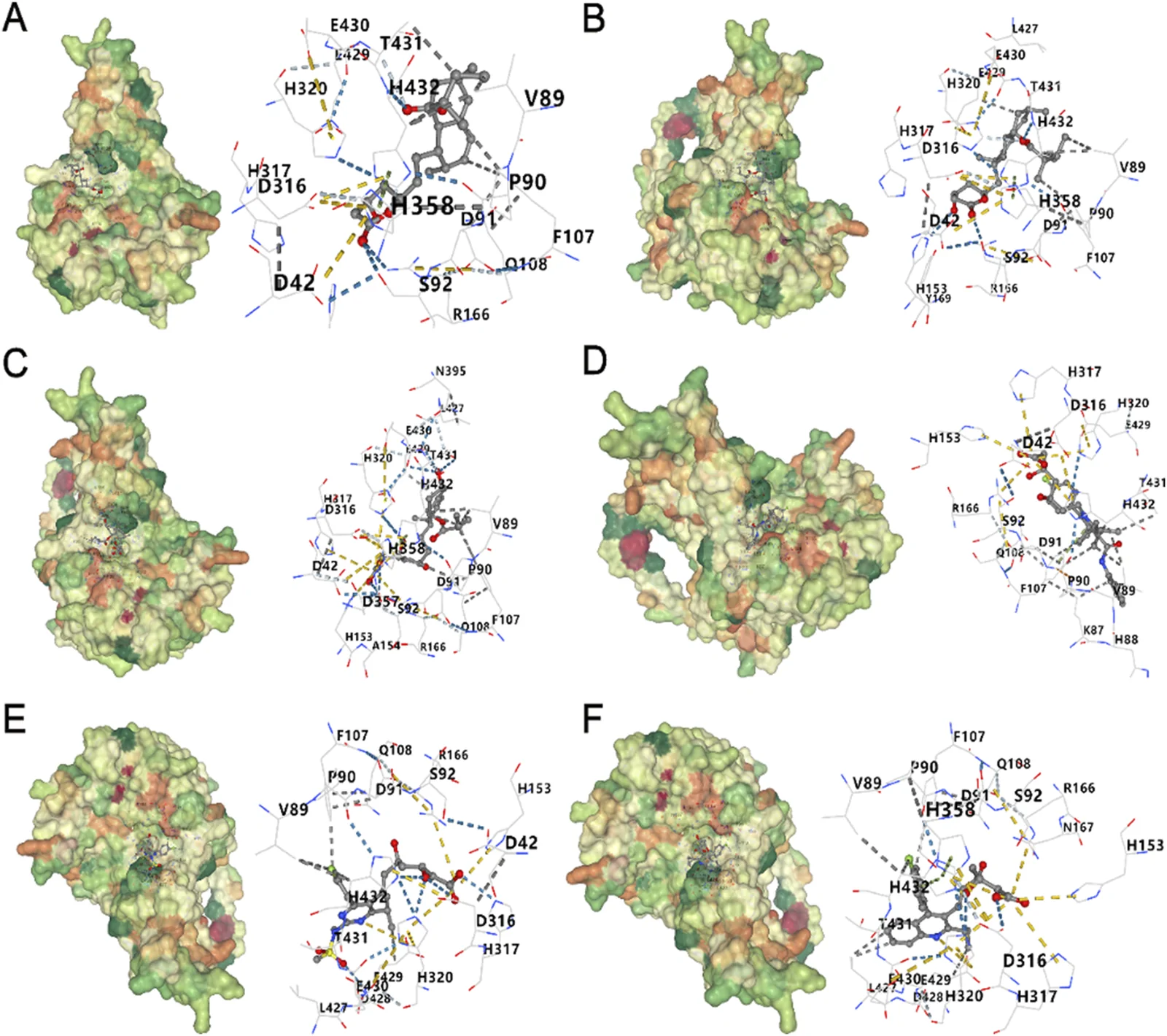
Molecular docking of ALPP and various statin drugs. The molecular docking results between protein ALPP and six statin compounds—Lovastatin **(A)**, Simvastatin **(B)**, Pravastatin **(C)**, Atorvastatin **(D)**, Rosuvastatin **(E)**, and Pitavastatin **(F)**—were visualized as shown in panels A–F. Each panel sequentially presents the global docking overview (left) and interaction diagram (right). The protein surface exhibits gradient coloration from green, orange, to red, reflecting the transition of amino acid properties from hydrophilic to hydrophobic characteristics. Key molecular interactions are denoted by distinct dashed lines: hydrogen bonds (blue), weak hydrogen bonds (light blue), hydrophobic interactions (gray), ionic bonds (yellow), π-π conjugation (green), and cation-π interactions (orange).

All six statins occupied the conserved binding pocket within ALPP, anchored by a shared set of contact residues. Complex stability depended on multiple intermolecular forces acting in concert: hydrogen bonding (classical and weak), hydrophobic contacts, π–π stacking, cation–π interactions, and ionic bonds. No single force type dominated. These docking results support structural complementarity between statins and ALPP, but static docking alone cannot account for conformational dynamics. We therefore turned to molecular dynamics simulations and binding free energy analysis to test whether these complexes hold up under physiological conditions.

### 3.8 Molecular dynamics simulation and binding free energy analysis

We ran molecular dynamics (MD) simulations to assess the dynamic stability and binding behavior of each protein–ligand complex. System stability and residue-level flexibility were analyzed first (Supplementary Figure 2). RMSD trajectories for both the wild-type protein and all complexes converged after ∼40 ns, remaining below 3 Å for the rest of the simulation (Supplementary Figure 2A). RMSF profiles across the core binding region (residues 23–481) tracked closely with the wild-type—the only stretches showing elevated fluctuation mapped to the N-terminal signal peptide (Supplementary Figure 2C).

We then asked whether ligand binding altered the overall protein fold. Radius of gyration (Rg) trajectories for the complexes were nearly indistinguishable from that of the unbound wild-type, ruling out any large-scale compaction or expansion upon binding (Supplementary Figure 2B). Solvent-accessible surface area (SASA) values told the same story: ligand-bound structures maintained surface exposure comparable to the wild-type, so the global conformation was preserved (Supplementary Figure 2D). When we superimposed the 0 ns and 100 ns snapshots, binding pockets remained structurally intact—with the exception of lovastatin. These results suggest that the ligands primarily influence local stability rather than inducing significant global conformational changes.

Structural and energetic analyses pointed to a defined binding mechanism. Frame superposition and Porcupine plots (Figures 5B,C) were consistent with a “lock-and-key” model: the binding pocket showed minimal deformation over the trajectory. Hydrogen bond occupancy analysis (Supplementary Figure 3) indicated that persistent H-bond contacts were a major stabilizing force in each complex. Free energy landscapes (FEL) for each system exhibited well-defined energy minima (blue regions in Figure 5A), indicating that the simulated systems sampled relatively low-energy conformational states. Furthermore, per-residue free energy decomposition highlighted specific residues lining the pocket that provided substantial favorable contributions to ligand anchoring (Figure 6A).

**FIGURE 5:**
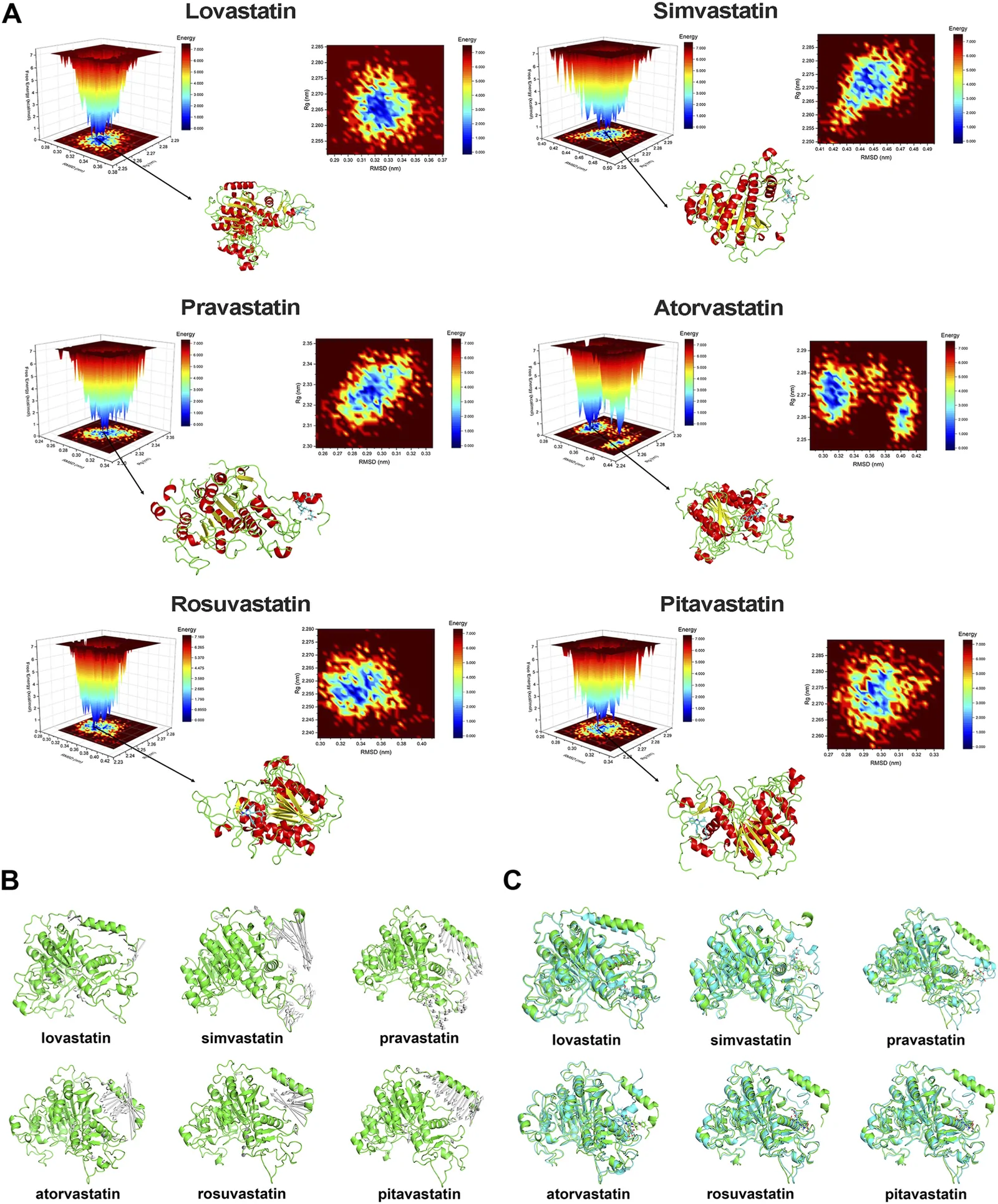
Thermodynamic stability and conformational dynamics analysis of the protein-statin complexes. **(A)** Gibbs Free Energy Landscapes (FEL) calculated using RMSD (x-axis) and Rg (y-axis) as reaction coordinates. The color scale represents the free energy level (kJ/mol), where dark blue areas indicate the global energy minima (lowest free energy conformations). The representative 3D structures extracted from these stable basins are displayed below each plot. **(B)** Porcupine plots visualizing the dominant motions of the protein backbone. The white cones (vectors) represent the direction and magnitude of residue displacement from the initial (0 ns) to the final (100 ns) conformation. **(C)** Structural superposition of the initial (0 ns, cyan) and final (100 ns, green) snapshots. The high structural overlap in the core domain indicates that the binding pocket undergoes no significant conformational changes upon ligand binding.

**FIGURE 6:**
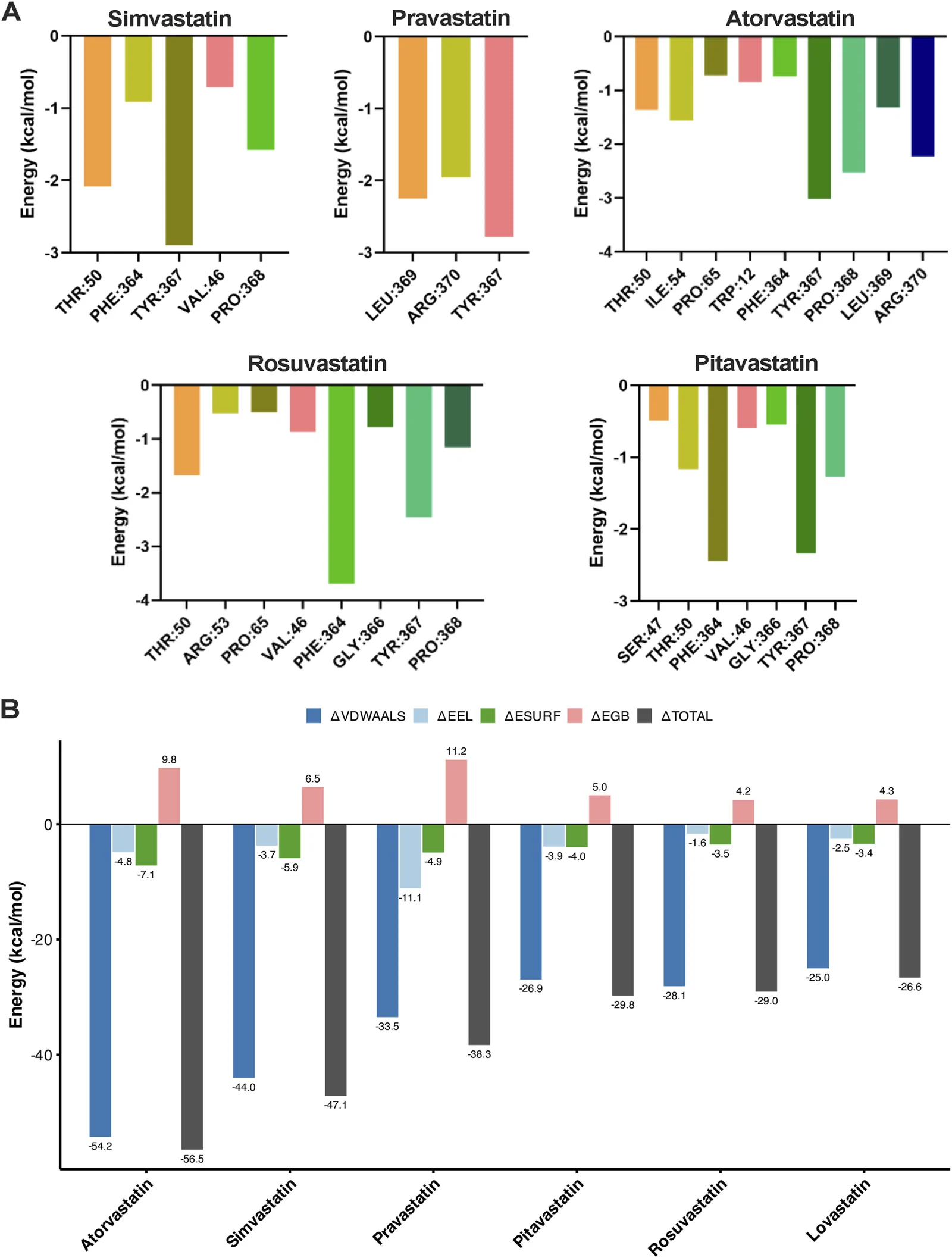
Thermodynamic profiling of statin-ALPP complexes reveals key interacting residues and energetic drivers. **(A)** Per-residue free energy decomposition highlighting the primary amino acid residues contributing to the binding affinity (kcal/mol) between ALPP and five statins (Simvastatin, Pravastatin, Atorvastatin, Rosuvastatin, and Pitavastatin). Negative energy values indicate favorable energetic contributions to the complex stability. Lovastatin was excluded from the residue-level decomposition because it did not maintain a sufficiently stable binding configuration during the simulation. **(B)** Binding free energy components for six statin-ALPP complexes calculated using the MM/GBSA (Molecular Mechanics/Generalized Born Surface Area) method. The bar graph details the thermodynamic driving forces: van der Waals interactions ( Δ VDWAALS), electrostatic energy ( Δ EEL), non-polar solvation free energy ( Δ ESURF), polar solvation free energy ( Δ EGB), and the overall total binding free energy ( Δ TOTAL).

Finally, to quantitatively assess the binding affinity, we calculated the binding free energies using the MM/GBSA method based on the stable trajectories of the final 10 ns (Figure 6B). Among the screened compounds, atorvastatin showed the most favorable calculated binding free energy with a binding free energy of −56.46 kcal/mol. The compounds with the next most favorable calculated values were simvastatin, pravastatin, and pitavastatin. Consequently, these four compounds were identified as potential candidates.

### 3.9 High expression of ALPP in hypertrophied ligamentum flavum correlates with lipid accumulation and fibrosis

To investigate the potential involvement of ALPP in Ligamentum flavum (LF) hypertrophy, we compared non-hypertrophied LF tissues from patients with LDH and hypertrophied LF (HLF) tissues. Western blot analysis revealed a striking upregulation of fibrosis-related markers (α-SMA, COL1, COL3, and FN1) in the HLF group compared to the LF group (Figure 7A). Concurrently, we observed a significant enrichment of ALPP protein in HLF tissues, which precisely coincided with the elevated expression of core lipid metabolism and deposition markers (ACC1, FABP4, FASN, and PLIN2) (Figure 7B).

**FIGURE 7:**
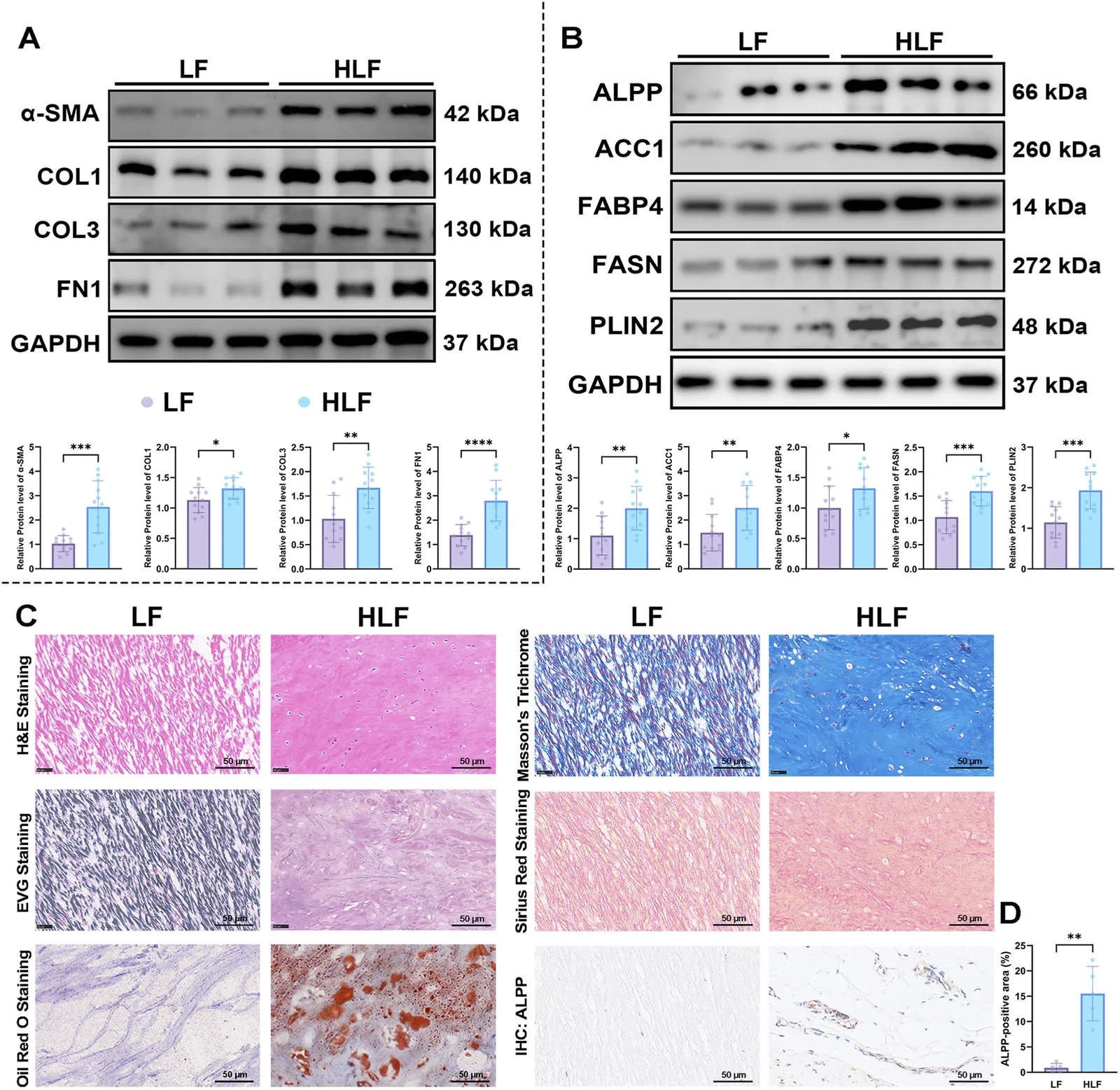
ALPP is highly expressed in clinical hypertrophied ligamentum flavum (HLF) and correlates with exacerbated fibrosis and ectopic lipid accumulation. **(A)** Western blot analysis and quantification of fibrosis-related extracellular matrix proteins in clinical normal (LF) and hypertrophied (HLF) ligamentum flavum tissues. **(B)** Western blot analysis and quantification of ALPP and key lipid metabolism markers in LF and HLF tissues. **(C)** Histological and immunohistochemical characterizations of LF and HLF tissues. Stains include H&E, EVG, Oil Red O, Masson’s trichrome, Sirius Red, and immunohistochemistry (IHC) for ALPP. Scale bars, 50 μm. **(D)** Quantification of ALPP immunohistochemical staining based on the percentage of DAB-positive area relative to the total tissue area. Data are mean ± SD. *P* < 0.05, **P < 0.01, ***P < 0.001, ****P < 0.0001.

To further evaluate the histopathological alterations, we performed a series of histological staining. H&E, EVG, Masson’s trichrome, and Sirius Red staining collectively demonstrated structural disorganization, fragmented elastic fibers, and dense collagen extracellular matrix accumulation in the HLF tissues (Figure 7C). Moreover, Oil Red O staining revealed a pervasive and abnormal increase in lipid accumulation within the HLF group. Finally, immunohistochemical (IHC) staining mapped the distribution of ALPP, confirming that ALPP was enriched *in situ* within the hypertrophic fibrotic area (Figure 7C). Quantitative analysis further showed that the ALPP-positive area was increased in the HLF group compared with the LF group (Figure 7D). In conclusion, these clinical tissue-based data provide compelling evidence that ALPP expression correlates with fibrosis and lipid accumulation during the progression of LF hypertrophy.

Given the high sequence similarity among alkaline phosphatase family members, we further evaluated the specificity of ALPP detection in LF cells. Two independent ALPP-targeting siRNAs significantly reduced ALPP mRNA expression, the approximately 66-kDa immunoreactive band, and ALPP immunofluorescence (Supplementary Figure 4). These concordant findings support the specificity of ALPP detection in LF cells.

### 3.10 Establishment of an *in vitro* FFA-induced lipid accumulation and fibrosis model, and evaluation of statin efficacies

To explore the regulatory role of lipid metabolism in LF cells, we simulated a lipotoxic microenvironment by treating LF cells with free fatty acids (FFA). Cell viability assays (CCK-8) established 200 µM as the maximal non-toxic FFA concentration for subsequent assays (Figure 8A). Nile Red staining (Figures 8B,C) and Oil Red O staining (Figures 8D,E) confirmed that 200 µM FFA robustly induced intracellular lipid droplet accumulation. RT-qPCR analyses further validated that FFA stimulation significantly upregulated the transcription of *ALPP*, lipogenic enzymes (*FASN, ACC1*), and the fibrotic marker *ACTA2* (Figure 8F). Thus, 200 µM FFA was established as the optimal dose for pathological modeling.

**FIGURE 8:**
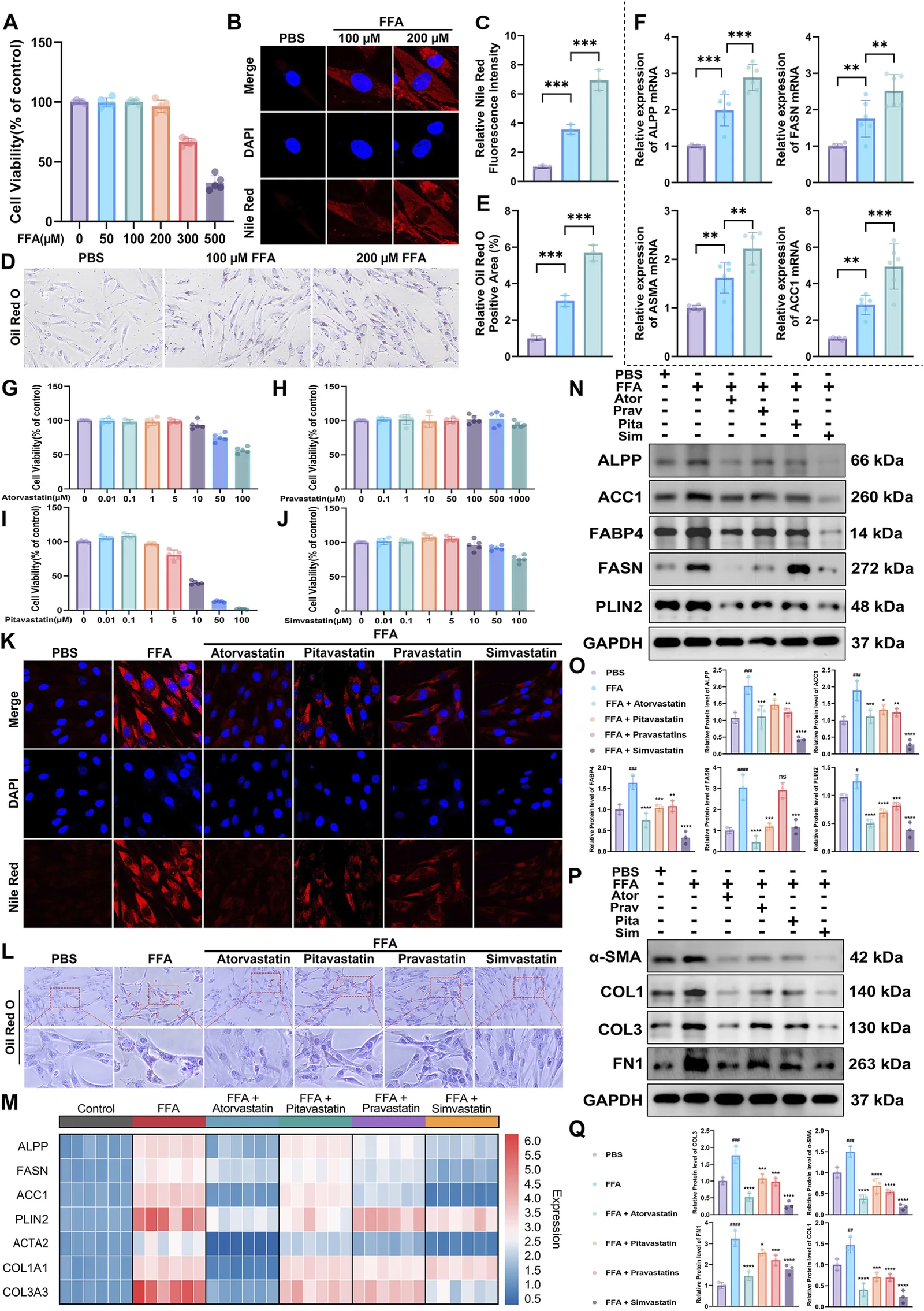
Statins alleviate FFA-induced lipid accumulation and fibrosis *in vitro*. **(A)** LF cell viability under FFA treatment (0–500 μM). **(B)** Representative Nile Red staining and **(C)** relative fluorescence quantification assessing lipid droplet accumulation in FFA-stimulated LF cells. **(D)** Representative Oil Red O staining and **(E)** quantification in LF cells. **(F)** RT-qPCR analysis of ALPP, lipogenic, and fibrotic genes. **(G)** Cytotoxicity profile of Atorvastatin, **(H)** Pravastatin, **(I)** Pitavastatin, and **(J)** Simvastatin. **(K)** Nile Red and **(L)** Oil Red O staining assessing statin-mediated suppression of lipid accumulation. **(M)** Transcriptomic heatmap of gene expression across statin treatments. **(N)** Western blot analysis and **(O)** quantification of ALPP and lipogenic proteins. **(P)** Western blot analysis and **(Q)** quantification of fibrotic proteins in LF cells following statin interventions. Data are mean ± SD. P < 0.05, **P < 0.01, ***P < 0.001, ****P < 0.0001.

Next, to screen for the most effective pharmacological interventions, we first evaluated the cytotoxicity profiles of four statins (Atorvastatin, Pravastatin, Pitavastatin, and Simvastatin) to determine their safe working concentrations (Figures 8G–J). Following co-treatment with FFA and the respective statins, morphological assessments via Nile Red (Figure 8K) and Oil Red O staining (Figure 8L) demonstrated that all four statins attenuated cellular lipid accumulation, with Atorvastatin and Simvastatin exhibiting the most profound reversal effects. Western blot analyses revealed that Atorvastatin and Simvastatin most potently suppressed the FFA-driven overexpression of ALPP and lipid metabolism proteins (ACC1, FABP4, FASN, PLIN2) (Figures 8N,O), as well as fibrotic matrix proteins (α-SMA, COL1, COL3, FN1) (Figures 8P,Q). Consistently, RT-qPCR analyses confirmed that both statins significantly downregulated the corresponding mRNA expression levels of these lipogenic and fibrotic genes (*ALPP, FASN, ACC1, PLIN2, ACTA2, COL1A1, COL3A1*) (Figure 8M). Based on their pharmacological efficacies in this screening, Atorvastatin and Simvastatin were selected as the primary candidates for downstream mechanistic experiments.

### 3.11 ALPP contributes to the protective effects of statins against FFA-induced lipid accumulation and fibrosis in LF cells

To evaluate whether statins engage ALPP in LF cells, we performed cellular thermal shift assays (CETSA). The data demonstrated that co-incubation with Simvastatin (Figures 9A,B) or Atorvastatin (Figures 9C,D) markedly enhanced the thermal stability of the ALPP protein across a temperature gradient (37 °C–62 °C), inducing a distinct rightward shift in its degradation curve. These results support target engagement between these statins and ALPP.

**FIGURE 9:**
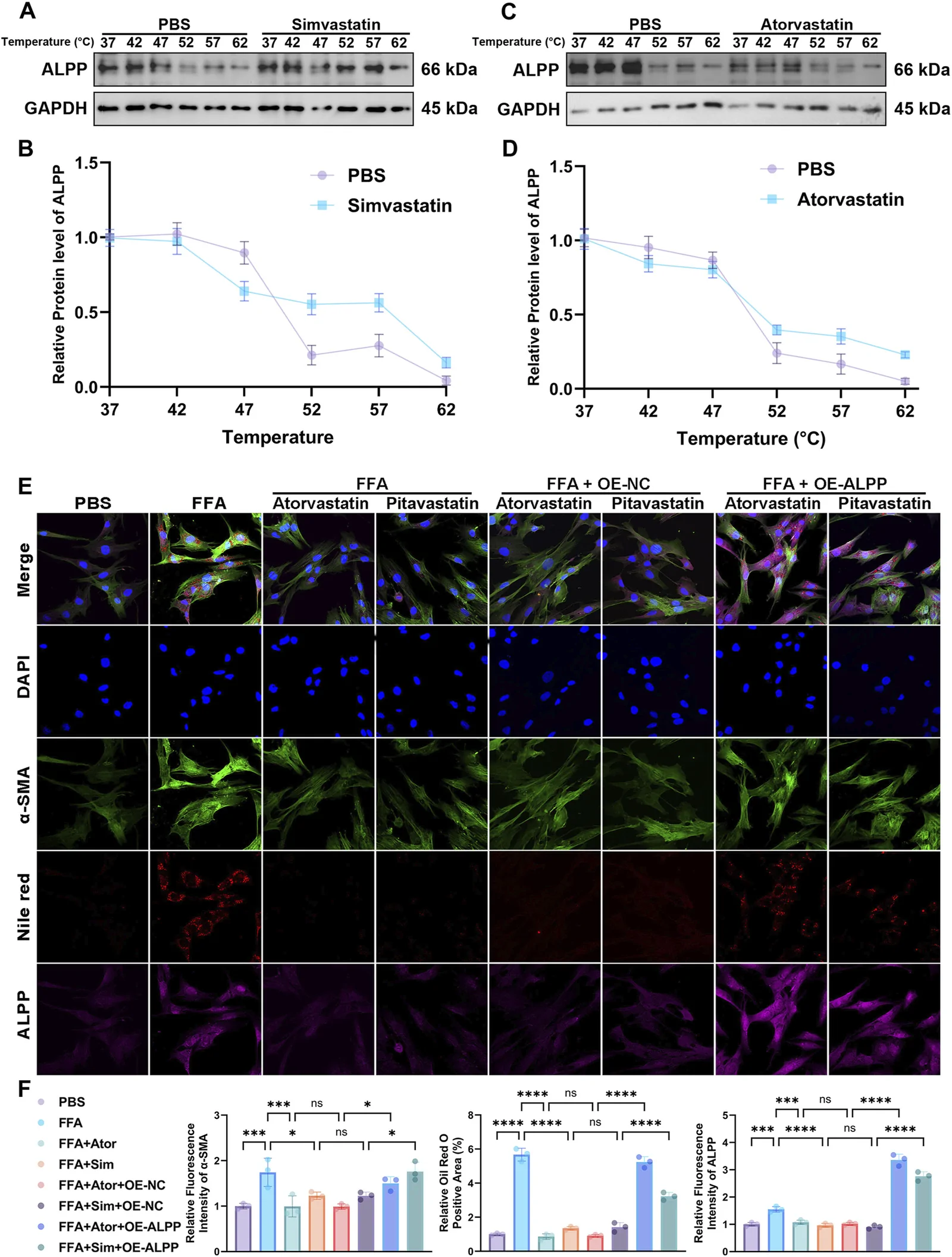
Statins engage ALPP, and ALPP overexpression attenuates their protective effects. **(A)** Cellular Thermal Shift Assay (CETSA) and **(B)** corresponding melting curves evaluating ALPP thermal stability following simvastatin treatment. **(C)** CETSA and **(D)** corresponding melting curves evaluating ALPP thermal stability following atorvastatin treatment. **(E)** Representative immunofluorescence co-staining of α-SMA, Nile Red, and *ALPP* in LF cells across treatments, with or without *ALPP* overexpression (*OE-ALPP*). **(F)** Quantification of fluorescence intensity and Oil Red O positive area. Data are mean ± SD. *P* < 0.05, ***P < 0.001, ****P < 0.0001; ns, not significant.

We next asked whether ALPP contributes to the protective effects of statins by performing rescue experiments with an ALPP overexpression plasmid (*OE-ALPP*). Immunofluorescence showed that Atorvastatin and Simvastatin each reversed FFA-induced myofibroblast activation (α-SMA) and lipid accumulation (Nile Red) whereas co-transfection with *OE-ALPP* counteracted both protective effects (Figures 9E,F). Western blot analyses further confirmed these findings: the statin-mediated repression of lipogenic proteins (ACC1, FABP4, FASN, PLIN2) (Figures 10A,B) and fibrotic drivers (α-SMA, COL1, COL3, FN1) (Figures 10C,D) was attenuated by *OE-ALPP*, restoring pathological expression to levels comparable to the FFA-alone group.

**FIGURE 10:**
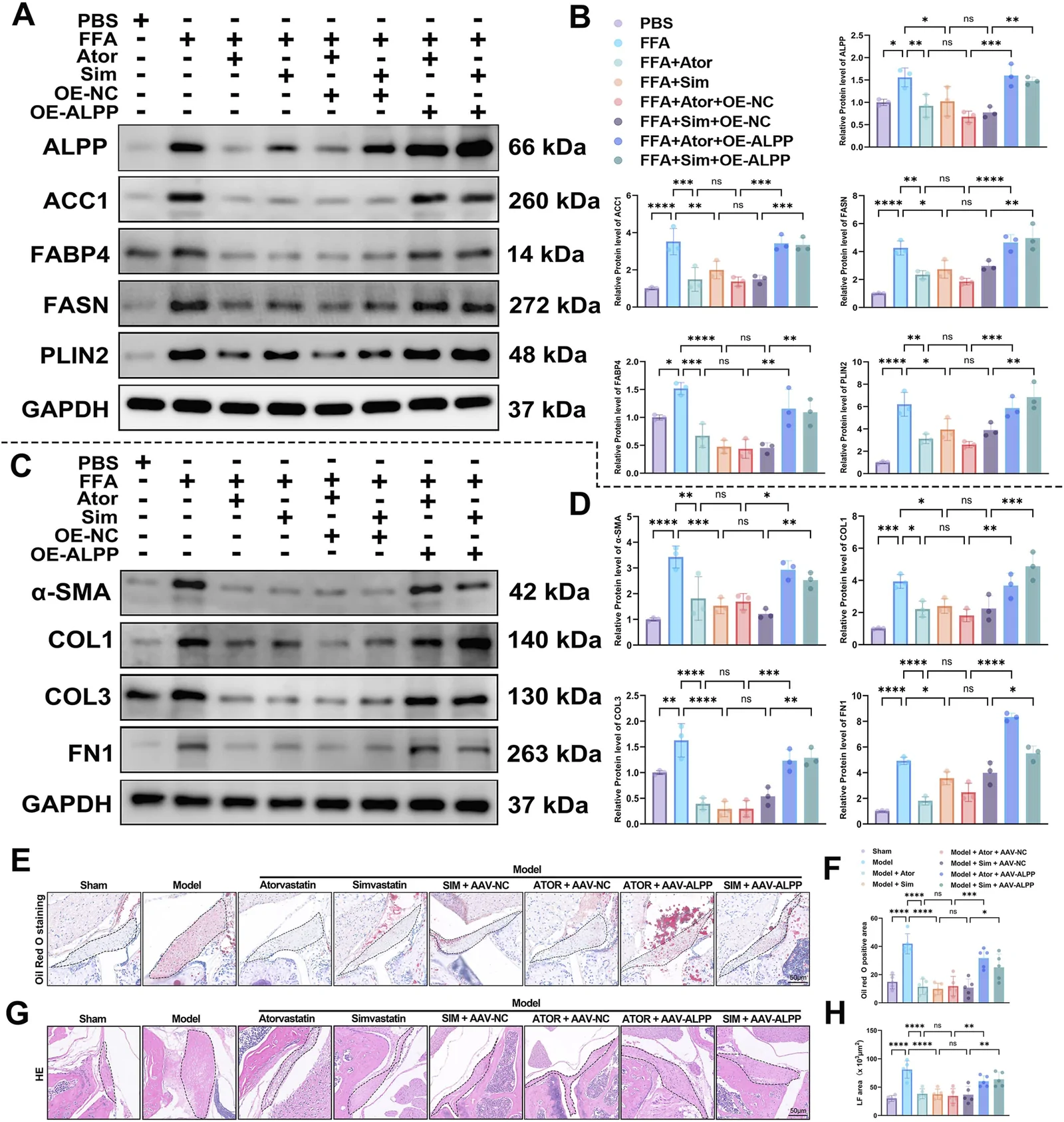
*In vitro* and *in vivo* evidence for the contribution of *ALPP* to the protective effects of statins. **(A)** Western blot analysis and **(B)** quantification of *ALPP* and lipogenic proteins in rescue experiments involving statins and *OE-ALPP*. **(C)** Western blot analysis and **(D)** quantification of fibrotic proteins. **(E)** Representative Oil Red O staining and **(F)** quantification of lipid area in LF tissues from an LSS mouse model following statin therapy and AAV-mediated ALPP overexpression. **(G)** Representative H&E staining and **(H)** quantification of LF cross-sectional area. Scale bars, 50 μm. Data are mean ± SD. *P* < 0.05, **P < 0.01, ***P < 0.001, ****P < 0.0001. (Sham: non-loaded controls; Model: bipedal-loading LF hypertrophy; AAV-NC: empty-vector control).

To determine whether these findings could be reproduced *in vivo*, we established a mouse model of LF hypertrophy and included matched AAV negative-control (AAV-NC) groups to distinguish ALPP-specific effects from those associated with viral delivery (Sham = non-loaded controls; Model = bipedal-loading LF hypertrophy; AAV-NC = empty-vector control). Histological assessment using H&E and Oil Red O staining showed that systemic administration of atorvastatin or simvastatin significantly reduced LF thickening and local lipid accumulation compared with the untreated model group. Administration of AAV-NC did not significantly affect the protective effects of either statin. In contrast, AAV-mediated ALPP overexpression significantly increased LF thickness and lipid deposition compared with the corresponding statin plus AAV-NC group, thereby attenuating the protective effects of atorvastatin and simvastatin (Figures 10E–H). Immunofluorescence analyses showed that the expression of COL1, COL3, α-SMA, COL2, SOX9, and RUNX2 was increased in the Model group compared with the Sham group (Figure 11). Atorvastatin and simvastatin reduced the expression of these markers, whereas AAV-mediated ALPP overexpression largely reversed these effects. These results further support the involvement of ALPP in fibrotic and chondro-osteogenic remodeling of the hypertrophied LF. Together with the *in vitro* rescue experiments, these results indicate that ALPP contributes to statin-mediated attenuation of lipid accumulation, fibrotic remodeling, and LF hypertrophy (Figure 12).

**FIGURE 11:**
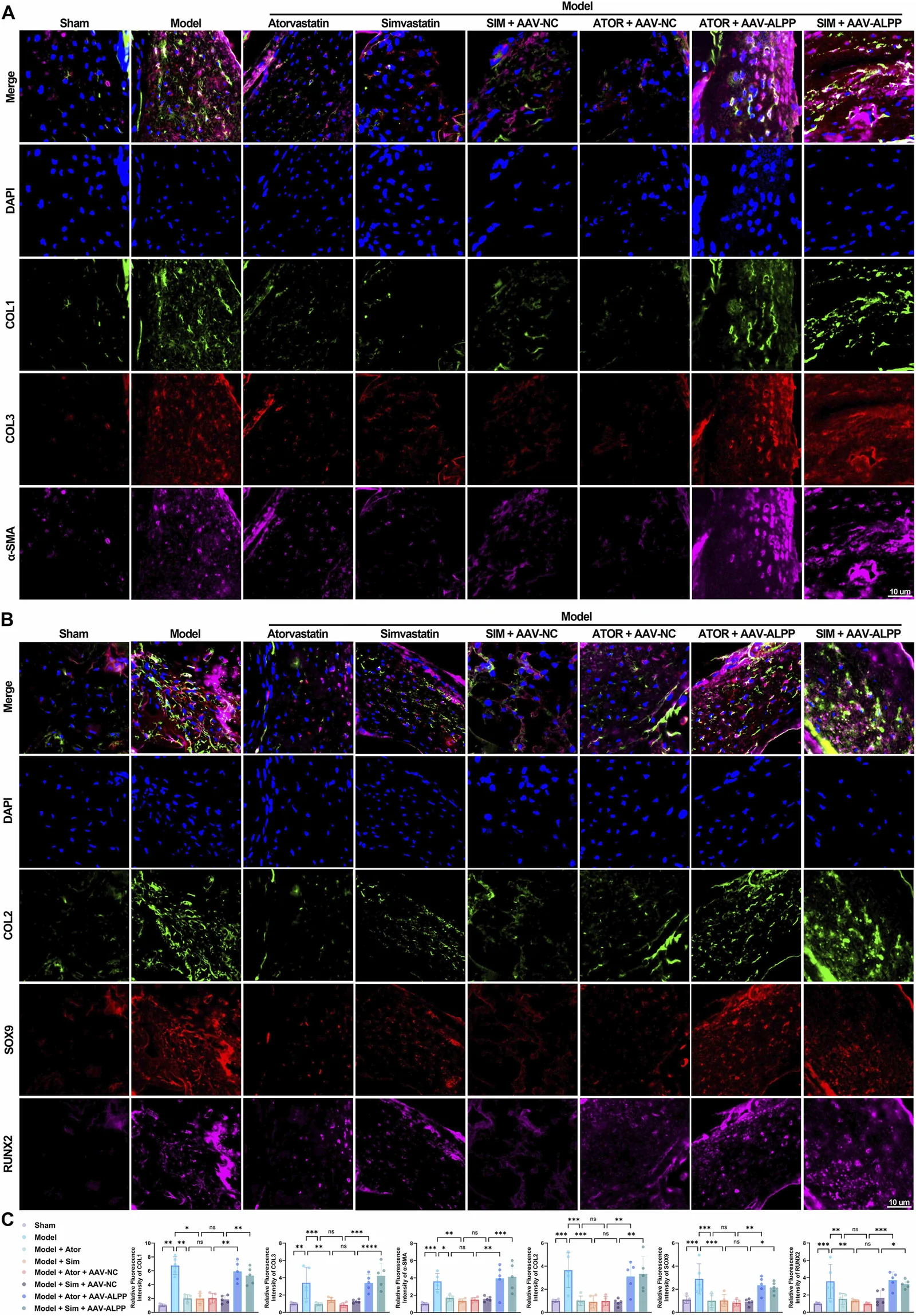
ALPP overexpression attenuates the inhibitory effects of statins on fibrotic and chondro-osteogenic remodeling in hypertrophied ligamentum flavum. **(A)** Representative immunofluorescence images of COL1, COL3, and α-SMA in ligamentum flavum tissues from the indicated groups. Nuclei were counterstained with DAPI. **(B)** Representative immunofluorescence images of COL2, SOX9, and RUNX2. **(C)** Quantification of the relative fluorescence intensities of COL1, COL3, α-SMA, COL2, SOX9, and RUNX2. Data are presented as the mean ± SD (n = 5 per group). Scale bars, 10 μm *P < 0.05, **P < 0.01, ***P < 0.001; ns, not significant. (Sham: non-loaded controls; Model: bipedal-loading LF hypertrophy; AAV-NC: empty-vector control).

**FIGURE 12:**
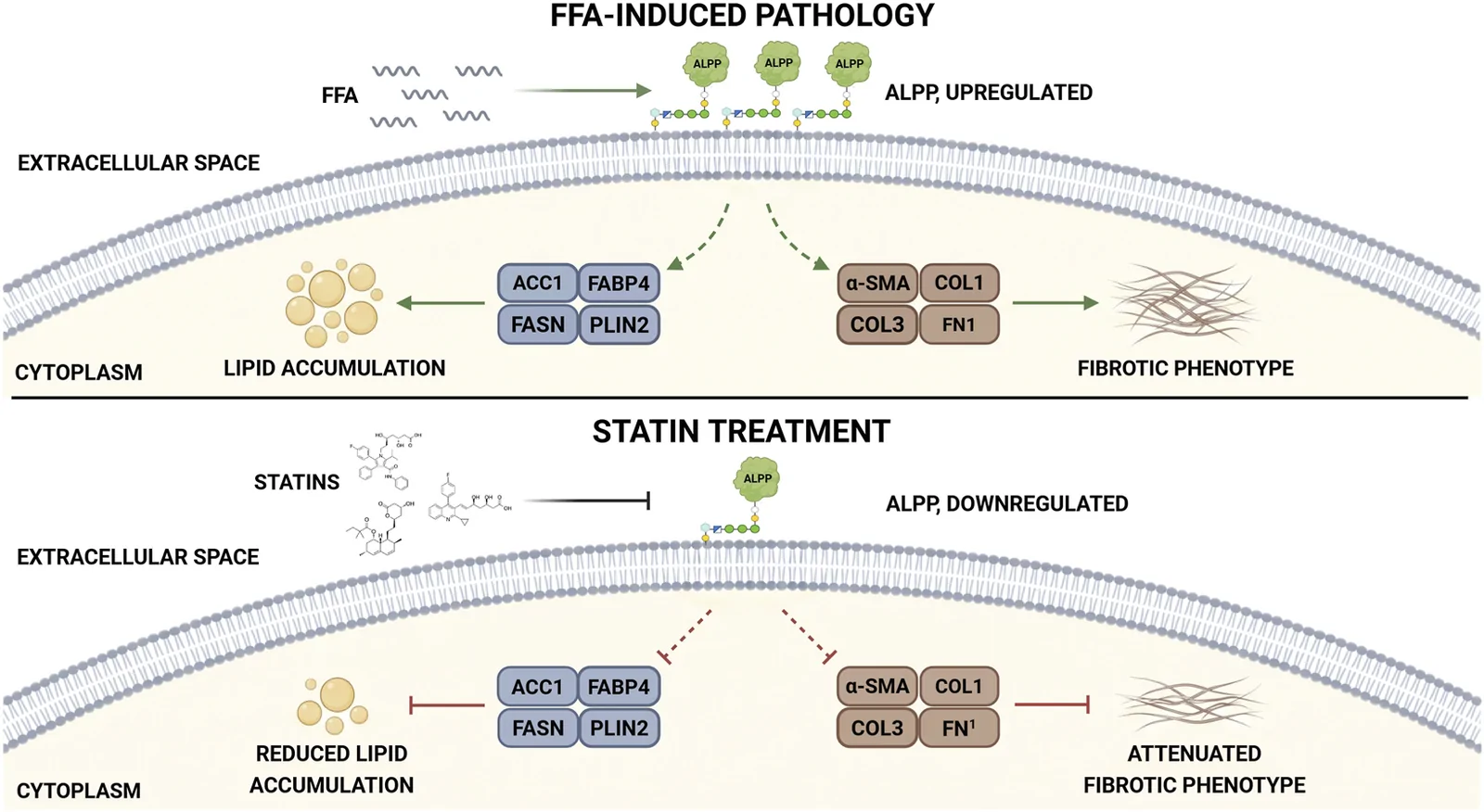
Overview of the molecular mechanisms by which statins alleviate LSS.

## 4 Discussion

LSS ranks among the most common degenerative spinal conditions, and thickening of the ligamentum flavum (LF) is its defining structural feature. Despite this, treatment options have not moved far beyond symptom management (Fang et al., 2024). Our study combined Mendelian randomization (MR), molecular docking, molecular dynamics simulations (MD), and both *in vitro* and *in vivo* experiments to investigate the potential role of placental alkaline phosphatase (ALPP) as a genetically supported risk factor for LSS. The genetic analyses supported a potential association between ALPP and LSS, while the experimental results suggested that ALPP may promote LF hypertrophy and fibrosis by enhancing lipid accumulation. Statins interacted with ALPP in our experimental models, and the overexpression experiments indicated that ALPP may contribute to their anti-lipogenic and anti-fibrotic effects.

Prior work on ALPP has focused mainly on systemic phosphate metabolism and bone mineralization. Our data suggest an additional potential role in LSS. MR and Bayesian colocalization analyses provided genetic support for an association between ALPP locus and LSS. However, these findings do not establish ALPP itself as a causal protein. In particular, colocalization indicates that ALPP-related pQTL and LSS association signals may share a causal variant or local genetic basis, but it does not establish the underlying biological mechanism. Histological evaluation of clinical specimens further showed that ALPP was upregulated in hypertrophic LF tissues relative to non-hypertrophic controls. Within these tissues, ALPP localized to fibrotic regions and showed spatial overlap with lipid metabolism markers, including ACC1, FABP4, and FASN. Together, these findings suggest that ALPP may contribute to a local lipotoxic microenvironment characterized by ectopic lipid accumulation and fibrotic remodeling, although this mechanism requires further functional validation.

The unit MR effect estimate for ALPP was small (OR approximately 1.001). This estimate should therefore be interpreted primarily as evidence supporting the direction of the genetically predicted association and the prioritization of ALPP for further investigation, rather than as an estimate of the expected clinical effect of targeting ALPP. Its biological interpretation also depends on the measurement scale of the protein exposure and the magnitude of change that can be achieved through intervention. Multivariable MR (MVMR) suggested that the association between ALPP and LSS remained after adjustment for obesity and diabetes. Nevertheless, MVMR cannot completely exclude residual pleiotropy, measurement error, or other unmeasured pathways. In addition, the phenome-wide association study (PheWAS) identified no adverse traits associated with ALPP at the phenome-wide significance threshold. Although this finding did not reveal major genetic safety signals, it should not be interpreted as evidence that therapeutic targeting of ALPP is safe. The biological relevance, targetability, and safety of ALPP require further experimental and clinical evaluation.

Our genetic mediation analysis suggested that selected immune-cell traits may partially contribute to the association between ALPP and LSS. This observation is consistent with previous evidence linking ectopic lipid accumulation to inflammatory responses. However, because immune-cell infiltration and inflammatory signaling were not directly assessed, these findings should be considered exploratory rather than evidence of an established immune-mediated mechanism.

Although statins are best known as HMG-CoA reductase (HMGCR) inhibitors, their pleiotropic effects on the musculoskeletal system have received increasing attention (Chamani et al., 2021; Zahedipour et al., 2022; Svec and Adameova, 2023). In this study, molecular dynamics simulations predicted stable statin–ALPP interactions, while cellular thermal shift assays supported ALPP target engagement by atorvastatin and simvastatin. Both statins attenuated FFA-induced lipid accumulation and fibrosis in LF cells and reduced LF hypertrophy in mice. These protective effects were counteracted by plasmid-mediated ALPP overexpression *in vitro* and AAV-mediated ALPP overexpression *in vivo*, indicating that ALPP contributes to statin action. However, our findings do not exclude the canonical HMGCR/mevalonate pathway, through which reductions in mevalonate-derived intermediates and protein prenylation may also exert anti-lipogenic and anti-fibrotic effects. The ALPP-associated mechanism may therefore operate alongside or interact with the canonical pathway, and their relative contributions remain to be determined. Given their established pharmacokinetics and long-term clinical safety profiles for approved indications, statins may represent candidates for drug-repurposing research in LSS (Dolivo et al., 2023; Sharpton and Loomba, 2023; Newman et al., 2019). However, their efficacy and safety for LSS cannot be inferred from the present study and require further preclinical and clinical evaluation.

Nevertheless, several limitations of this study should be acknowledged. First, the genomic data utilized for the MR and PheWAS analyses were predominantly derived from populations of European ancestry. Validation in populations with diverse ancestries is necessary to assess the generalizability of the association between ALPP and LSS. The degree of participant overlap between the proteomic exposure datasets and the UK Biobank LSS outcome dataset could not be fully determined from the available summary-level information. Although only instruments with F-statistics >10 were retained to reduce weak-instrument bias, the potential influence of sample overlap could not be eliminated. Second, the *in vitro* FFA-induced lipid accumulation model and the *in vivo* mechanical stress model primarily capture the acute phases of cellular stress and tissue remodeling (Cao et al., 2023b; Zheng et al., 2021). They may not fully recapitulate the chronic, progressive nature of human LSS, which typically develops over decades through complex biomechanical and epigenetic interactions (Zhao et al., 2024). Third, immune-cell mediation was inferred genetically but was not validated using direct immune-cell markers or inflammatory/cytokine profiling. These findings therefore remain exploratory and require experimental confirmation. Fourth, we did not distinguish ALPP-associated statin effects from those mediated by the canonical HMGCR/mevalonate pathway. Further pathway-specific rescue and genetic experiments are required to determine their relative contributions. Fifth, the clinical tissue analysis also has several limitations. Because LF tissue cannot ethically be obtained from healthy individuals, non-hypertrophied LF tissues from patients undergoing surgery for LDH were used as reference samples. These patients may nevertheless have local degenerative, inflammatory, or mechanical changes and therefore do not represent truly healthy controls. In addition, all participants were selected from a surgical population, which may introduce surgical selection bias. The LSS group was also older and had a higher BMI than the LDH group, and these differences may have confounded the observed tissue-level associations. Although statin use did not differ significantly between groups, residual confounding related to medication exposure cannot be excluded. Additionally, although short-term cell viability was not visibly compromised under the current experimental conditions, the long-term effects of prolonged FFA and statin exposure on LF-cell viability were not evaluated. Long-term cytotoxicity cannot be completely excluded and should be systematically investigated in future studies. Finally, micro-CT analysis was not performed in the present study; therefore, whether ALPP contributes to progressive LF mineralization or ossification remains to be determined.

## 5 Conclusion

In conclusion, by integrating genetic and experimental evidence, this study identifies ALPP as a genetically supported candidate to LSS and links it to local lipid accumulation, fibrosis, and LF hypertrophy. We further demonstrated that statins engage ALPP and attenuate these pathological changes in experimental models. Genetic mediation analyses also suggested possible immune-cell involvement, although this requires direct experimental validation. These findings support further evaluation of statins and specific ALPP inhibitors as potential therapeutic strategies for LSS.

## Data Availability

Publicly available datasets were analyzed in this study. This data can be found here: deCODE database (https://www.decode.com/); MRC IEU OpenGWAS database (https://gwas.mrcieu.ac.uk/, dataset IDs: finn-b-M13_SPINSTENOSIS and ukb-d-M13_SPINSTENOSIS); DrugBank (https://go.drugbank.com/); ChEMBL (https://www.ebi.ac.uk/); AstraZeneca PheWAS Portal (https://azphewas.com/); PheWeb (https://pheweb.org/).

## Glossary

**AAV**: Adeno-associated virus
**ACC1**: Acetyl-CoA carboxylase 1
**ALPP**: Placental alkaline phosphatase
**CCK-8**: Cell Counting Kit-8
**CETSA**: Cellular thermal shift assay
**CI**: Confidence interval
**EVG**: Elastic Van Gieson
**FABP4**: Fatty acid-binding protein 4
**FASN**: Fatty acid synthase
**FEL**: Free energy landscape
**FFA**: Free fatty acid
**FN1**: Fibronectin 1
**GWAS**: Genome-wide association study
**H&E**: Hematoxylin and eosin
**HEIDI**: Heterogeneity in dependent instruments
**HLF**: Hypertrophied ligamentum flavum
**HMGCR**: 3-Hydroxy-3-methylglutaryl-CoA reductase
**IHC**: Immunohistochemistry
**IVW**: Inverse variance weighted
**LD**: Linkage disequilibrium
**LF**: Ligamentum flavum
**LSS**: Lumbar spinal stenosis
**MD**: Molecular dynamics
**MM/GBSA**: Molecular mechanics/generalized Born surface area
**MR**: Mendelian randomization
**MVMR**: Multivariable Mendelian randomization
**OR**: Odds ratio
**PheWAS**: Phenome-wide association study
**PLIN2**: Perilipin 2
**pQTL**: Protein quantitative trait locus
**Rg**: Radius of gyration
**RMSD**: Root mean square deviation
**RMSF**: Root mean square fluctuation
**RT-qPCR**: Real-time quantitative polymerase chain reaction
**SASA**: Solvent-accessible surface area
SD: Standard deviation
**SMR**: Summary-data-based Mendelian randomization
**SNP**: Single nucleotide polymorphism
**TSA**: Tyramide signal amplification
**α-SMA**: Alpha-smooth muscle actin
