# Supplementary material for: Placental alkaline phosphatase promotes lipid accumulation and fibrosis in ligamentum flavum hypertrophy and represents a potential target for statin repurposing
Source: Front Pharmacol. 2026 Sep 29;17:1910904. doi: 10.3389/fphar.2026.1910904 (PMC13658204; doi:10.3389/fphar.2026.1910904)
Supplement: Supplementary file 1 [file Table3.docx]

Table S3 Antibody information

| **Antibodies human (mouse or rat)** | **Source** | **Cat No** | **RRID** |
| --- | --- | --- | --- |
| GAPDH | Proteintech | Cat No. 60004-1-Ig | AB_2107436 |
| RUNX2  SOX9  COL1  COL2  COL3  FN1  ALPP  ACC1  FABP4  FASN  PLIN2 | Proteintech  Proteintech  Proteintech  Proteintech  Proteintech  Proteintech  Proteintech  Proteintech  Proteintech  Proteintech  Proteintech | 82636-2-RR  67439-1-Ig  66761-1-Ig  86139-2-RR  68320-1-Ig  66042-1-Ig  60294-1-Ig  67373-1-Ig  67167-1-Ig  66591-1-Ig  60340-1-Ig | AB_3086495  AB_2882675  AB_2882107  AB_3744654  AB_2935393  AB_11182385  AB_2881409  AB_2882621  AB_2882463  AB_2881951  AB_2881449 |
