# Supplementary material for: Placental alkaline phosphatase promotes lipid accumulation and fibrosis in ligamentum flavum hypertrophy and represents a potential target for statin repurposing
Source: Front Pharmacol. 2026 Sep 29;17:1910904. doi: 10.3389/fphar.2026.1910904 (PMC13658204; doi:10.3389/fphar.2026.1910904)
Supplement: Supplementary file 2 [file Table4.docx]

Table S4 The primers sequencing was used in RT-qPCR

| **name** | **Forward primer** | **Reverse primer** |
| --- | --- | --- |
| ALPP | ATCGCTACGCAGCTCATCTC | CTGCACCAGATTCTTCCCGT |
| FASN | CCTGGCTGCCTACTACATCG | CACATTTCAAAGGCCACGCA |
| ACC1 | TAGTCTGCCACGGATCCAGA | GGGAGGGATCTCTGAGGGTT |
| PLIN2 | CACCCTCCTGTCCAACATCC | GTGAGGAGGCTGTCAGACAC |
| ACTA2 | GCCCTCCTTCATCGGTATGG | CGATCCCAGGGTACATCGTG |
| COL1A1 | CCTGGTGATGCTGGTGCTAA | ACATTACCAATGGGGCCAGG |
| COL3A3 | GAAAGGTGAAGGAGGCCCTC | GCCACGTTCACCTTTGACAC |
