# Supplementary material for: Placental alkaline phosphatase promotes lipid accumulation and fibrosis in ligamentum flavum hypertrophy and represents a potential target for statin repurposing
Source: Front Pharmacol. 2026 Sep 29;17:1910904. doi: 10.3389/fphar.2026.1910904 (PMC13658204; doi:10.3389/fphar.2026.1910904)
Supplement: Supplementary file 3 [file Table7.docx]

**Table S7** Heterogeneity test of Mendelian randomization analysis for Olink_UKB-derived proteins on lumbar spinal stenosis.

| exposure | outcome | Q | Q_df | Q_pval | I^2^(%) |
| --- | --- | --- | --- | --- | --- |
| BMP6 | SPINSTENOSIS | 41.23799 | 22 | 0.00772 | 46.65 |
| ALPP | SPINSTENOSIS | 85.28583 | 69 | 0.08916 | 19.1 |
| EPHB4 | SPINSTENOSIS | 14.0763 | 22 | 0.898749 | 0 |

Q: Cochran's Q test statistic; Q_df: degrees of freedom for Q test; Q_pval: P-value of Q test. I² statistic reflects the proportion of total variability attributed to heterogeneity among instrumental variables: I² ≤ 0, Defined as 0, indicating no observed heterogeneity. I² = 0–25%, Suggests mild heterogeneity. I² = 25–50%, Indicates moderate heterogeneity. I² > 50%, Implies substantial heterogeneity. Calculation formula: I^2^=(Q-df)/Q×100%

**Table S8** Heterogeneity test of Mendelian randomization analysis for SomaScan-derived proteins on lumbar spinal stenosis.

| exposure | outcome | Q | Q_df | Q_pval | I^2^(%) |
| --- | --- | --- | --- | --- | --- |
| ALPP | SPINSTENOSIS | 62.86408 | 53 | 0.166465 | 15.69 |

Q: Cochran's Q test statistic; Q_df: degrees of freedom for Q test; Q_pval: P-value of Q test. I² statistic reflects the proportion of total variability attributed to heterogeneity among instrumental variables: I² ≤ 0, Defined as 0, indicating no observed heterogeneity. I² = 0–25%, Suggests mild heterogeneity. I² = 25–50%, Indicates moderate heterogeneity. I² > 50%, Implies substantial heterogeneity. Calculation formula: I^2^=(Q-df)/Q×100%.

**Table S9** Horizontal Pleiotropy Assessment of Olink_UKB-Derived Proteins in Mendelian Randomization Analysis for Lumbar spinal stenosis

| exposure | outcome | egger_intercept | SE | pvalue |
| --- | --- | --- | --- | --- |
| BMP6 | SPINSTENOSIS | -0.00654 | 0.017095 | 0.7057 |
| ALPP | SPINSTENOSIS | -7.9E-05 | 6.89E-05 | 0.258032 |
| EPHB4 | SPINSTENOSIS | -7.4E-05 | 0.000133 | 0.581461 |

SE, Standard Error

**Table S10** Horizontal Pleiotropy Assessment of SomaScan-Derived Proteins in Mendelian Randomization Analysis for Lumbar spinal stenosis

| exposure | outcome | egger_intercept | SE | pvalue |
| --- | --- | --- | --- | --- |
| ALPP | SPINSTENOSIS | 0.00012 | 9.06E-05 | 0.192456 |

SE, Standard Error.

**Table S11** Steiger Directionality Test of Olink_UKB-Derived Proteins in Mendelian Randomization Analysis for Lumbar spinal stenosis

| exposure | outcome | snp_r2.exposure | snp_r2.outcome | correct_causal_direction | steiger_pvalue |
| --- | --- | --- | --- | --- | --- |
| BMP6 | SPINSTENOSIS | 0.033855 | 0.000543 | TRUE | 5.02E-214 |
| ALPP | SPINSTENOSIS | 0.521162 | 0.000292 | TRUE | 0 |
| EPHB4 | SPINSTENOSIS | 0.044414 | 7.36E-05 | TRUE | 0 |

SNP, single nucleotide polymorphism; r^2^, proportion of variance explained.

**Table S12** Steiger Directionality Test of SomaScan-Derived Proteins in Mendelian Randomization Analysis for Lumbar spinal stenosis

| exposure | outcome | snp_r2.exposure | snp_r2.outcome | correct_causal_direction | steiger_pval |
| --- | --- | --- | --- | --- | --- |
| ALPP | SPINSTENOSIS | 0.215757 | 0.000226 | TRUE | 0 |

SNP, single nucleotide polymorphism; r^2^, proportion of variance explained.

**Table S13** Results of the SMR analysis of the proteins for lumbar spinal stenosis

| exposure | topSNP | b_SMR | se_SMR | p_HEIDI |
| --- | --- | --- | --- | --- |
| BMP6 | rs9406052 | 0.001108 | 0.001249 | 3.26E-01 |
| ALPP | rs115624040 | 0.000347 | 0.000522 | 7.53E-01 |
| EPHB4 | rs314360 | 0.001871 | 0.0011 | 7.76E-01 |

SMR，Summary-data-based Mendelian Randomization; SNP; single nucleotide polymorphism; HEIDI，Heterogeneity in Dependent Instruments.

**Table S14** SMR Analysis of SomaScan-Derived Proteins for Lumbar spinal stenosis

| exposure | topSNP | b_SMR | se_SMR | p_HEIDI |
| --- | --- | --- | --- | --- |
| ALPP | rs12478529 | 0.000835 | 0.000671 | 8.01E-01 |

SMR，Summary-data-based Mendelian Randomization. SNP，single nucleotide polymorphism；HEIDI，Heterogeneity in Dependent Instruments.

**Table S15** Phenome-wide association study (PheWAS) results of protein and idiopathic pulmonary fibrosis.

| Exposure | Top p-value in gene | Phenotype |
| --- | --- | --- |
| ALPP | 9.80E-08 | Disorders of sacrum |
| ALPP | 9.80E-08 | Disorders of coccyx |
| ALPP | 1.30E-07 | Osteopenia or other disorder of bone and cartilage |
| ALPP | 1.90E-07 | Stomatitis and mucositis (ulcerative) |
| ALPP | 2.70E-07 | Congenital anomalies of skin |
| ALPP | 2.80E-07 | Rosacea |

**Table S16** Phenome-wide association study (PheWAS) results

| Exposure | p-value | Phenotype |
| --- | --- | --- |
| ALPP | 5.93E-07 | Chapter XIII Diseases of the musculoskeletal system and connective tissue |
| ALPP | 2.79E-05 | Chapter II Neoplasms |
| ALPP | 6.47E-05 | Chapter XIV Diseases of the genitourinary system |
| ALPP | 0.000208 | Chapter XI Diseases of the digestive system |
| ALPP | 0.000228 | Chapter I Certain infectious and parasitic diseases |
| ALPP | 0.000229 | Chapter VI Diseases of the nervous system |
| ALPP | 0.000263 | Chapter X Diseases of the respiratory system |
| ALPP | 0.000357 | Chapter V Mental and behavioral disorders |
| ALPP | 0.000359 | Chapter XXI Factors influencing health status and contact with health services |
| ALPP | 0.000494 | Chapter VII Diseases of the eye and adnexa |
| ALPP | 0.000755 | Chapter XVII Congenital malformations deformations and chromosomal abnormalities |
| ALPP | 0.001 | Chapter IX Diseases of the circulatory system |
| ALPP | 0.0016 | Chapter XV Pregnancy childbirth and the puerperium |
| ALPP | 0.0027 | Chapter III Diseases of the blood and blood-forming organs and certain disorders involving the immune mechanism |
| ALPP | 0.0027 | Chapter XVIII Symptoms signs and abnormal clinical and laboratory findings not elsewhere classified |
| ALPP | 0.0032 | Chapter IV Endocrine nutritional and metabolic diseases |
| ALPP | 0.0033 | Chapter VIII Diseases of the ear and mastoid process |
| ALPP | 0.004 | Chapter XII Diseases of the skin and subcutaneous tissue |
